# Possible rodent equivalent of the posterior cingulate cortex (area 23) interconnects with multimodal cortical and subcortical regions

**DOI:** 10.3389/fnins.2023.1194299

**Published:** 2023-06-13

**Authors:** Xiao-Jun Xiang, Sheng-Qiang Chen, Xue-Qin Zhang, Chang-Hui Chen, Shun-Yu Zhang, Hui-Ru Cai, Song-Lin Ding

**Affiliations:** ^1^Department of Psychology, School of Health Management, Guangzhou Medical University, Guangzhou, China; ^2^Key Laboratory of Neuroscience, School of Basic Medical Sciences, Guangzhou Medical University, Guangzhou, China; ^3^Allen Institute for Brain Science, Seattle, WA, United States

**Keywords:** cingulate cortex, retrosplenial cortex, orbital frontal cortex, postrhinal cortex, claustrum, anterior thalamic nucleus, connections, pulvinar

## Abstract

Posterior cingulate cortex (area 23, A23) in human and monkeys is a critical component of the default mode network and is involved in many diseases such as Alzheimer’s disease, autism, depression, attention deficit hyperactivity disorder and schizophrenia. However, A23 has not yet identified in rodents, and this makes modeling related circuits and diseases in rodents very difficult. Using a comparative approach, molecular markers and unique connectional patterns this study has uncovered the location and extent of possible rodent equivalent (A23~) of the primate A23. A23 ~ but not adjoining areas in the rodents displays strong reciprocal connections with anteromedial thalamic nucleus. Rodent A23 ~ reciprocally connects with the medial pulvinar and claustrum as well as with anterior cingulate, granular retrosplenial, medial orbitofrontal, postrhinal, and visual and auditory association cortices. Rodent A23 ~ projects to dorsal striatum, ventral lateral geniculate nucleus, zona incerta, pretectal nucleus, superior colliculus, periaqueductal gray, and brainstem. All these findings support the versatility of A23 in the integration and modulation of multimodal sensory information underlying spatial processing, episodic memory, self-reflection, attention, value assessment and many adaptive behaviors. Additionally, this study also suggests that the rodents could be used to model monkey and human A23 in future structural, functional, pathological, and neuromodulation studies.

## Introduction

The posterior cingulate gyrus in human and non-human primates (NHP) mainly contains the retrosplenial cortex (RS), which usually includes Brodmann’s areas 29 and 30 (A29 and A30, respectively), and the posterior cingulate cortex (PCC), which contains a large area 23 (A23) and a smaller area 31 (A31) (Brodmann, 1909; Vogt et al., 1987; Morecraft et al., 2004; Ding et al., 2016). Topographically, A30, A23 and A31 are located dorsal to A29, A30 and A23, respectively, and extend together as an arch around the splenium of the corpus callosum (CCS) (Vogt et al., 1987; Morris et al., 1999; Morecraft et al., 2004; Vogt et al., 2005). As in human and NHP, rodent A29 [granular RS (RSg)] is similarly located dorsal and caudal to the CCS while A30 [agranular RS (RSag)] is located dorsolateral to A29 (Sugar et al., 2011; Vogt and Paxinos, 2014). However, homologous PCC has not been reported in rodent literature so far. Instead, the region lateral to A30 has been treated as a lateral agranular region of the RS (RSagl, e.g., Swanson, 2004; Hu et al., 2020; Wang et al., 2020) or a part of the medial secondary visual cortex (area 18b or V2MM, e.g., Vogt and Miller, 1983; Sripanidkulchai and Wyss, 1986; van Groen et al., 1999; Paxinos and Franklin, 2012). The reasons for the difficulty of identifying the rodent equivalent of the PCC may include both historical and cytoarchitectonic aspects. Historically, the PCC has not been identified in lower mammals since Brodmann’s comparative mapping of the cerebral cortex (Brodmann, 1909; Vogt and Paxinos, 2014). Cytoarchitectonically, the existence (for the PCC) or lack (for the RS) of an inner granular layer 4 (L4) was used as the main criteria to define the PCC and RS, respectively (Brodmann, 1909; Morris et al., 1999; Vogt et al., 2005). Accordingly, the region located immediately lateral to A30 in rodents was not treated as the PCC since the region did not appear to have a L4 (Vogt and Paxinos, 2014).

However, in modern literature, brain structures/regions have also been defined using other important organizational features such as connectional patterns (Fudge et al., 2004; Gehrlach et al., 2020) and molecular signatures (Wallén-Mackenzie et al., 2020; Chen et al., 2022; Ding et al., 2022) as well as using combination of multimodal data (Ding and van Hoesen, 2010, 2015; Ding, 2022). In this study, we aim to investigate the connections of the region located immediately lateral to A30 in rat and mouse brains to determine whether this region possess similar connectional patterns as the monkey PCC (mainly A23) does. A23 but not adjoining A30 in the NHP was reported to receive strong inputs from the anteromedial thalamic nucleus (AM; Baleydier and Mauguiere, 1980; Vogt et al., 1987). Based on this finding we speculate that if the RSag (A30) and the RSagl (or part of it) in rodents receives, respectively, few or strong AM inputs, the latter region is likely the rodent equivalent of the monkey and human A23. Furthermore, if the possible rodent equivalent of A23 (termed A23 ~ in this study) identified based on the AM inputs is likely the homolog of the primate A23, A23 ~ should has similar brain-wide connectivity to that of the monkey A23 and should also be distinguishable from adjoining A30.

The results of the present study have confirmed above speculations and thus provided strong connectional evidence for the existence of possible rodent homolog of the primate A23. The brain-wide connections of A23 ~ revealed in this study would provide important insights into both evolutional and functional roles of A23~. Moreover, the identification of rodent A23 ~ also suggests that rodents can be used as animal models for future studies of the NHP and human PCC (mainly A23). In human, dysfunctions of the PCC have been observed in many neurological and mental diseases such as Alzheimer’s disease, traumatic brain injury, autism, depression, attention deficit hyperactivity disorder, addiction, and schizophrenia (Bluhm et al., 2009; Leech and sharp, 2014; Rolls, 2019; Zhang and Volkow, 2019). Detailed studies are needed in rodent models to gain more insights into the neural circuits and mechanisms of these diseases and the development of interference strategies.

## Materials and methods

### Animals

Forty-two adult Sprague–Dawley rats of both sexes weighing 280–310 g (Beijing Vital River Laboratory Animal Technology Co., Ltd., Beijing, China) were used in this study. All the animals were placed in the same environment with suitable temperature and controlled light, as well as free access to food and water. All surgery operations were performed under the deep anesthesia to alleviate their pain. All experimental procedures were followed in accordance with the protocols that have been approved by the Institutional Animal Care and Use Committee of Guangzhou Medical University.

### Surgery procedure and tracer injections in rats

The rats were randomly picked up for surgery without knowing sexes. Four to six animals were used for each injection region of interest (ROI) to secure at least three animals having correct ROI injections. The surgeries were carried out when the rats were deeply anesthetized with sodium pentobarbital (40 mg/kg, i. p.). The rat was fixed in a stereotaxic frame and the head hairs were shaved and a midline incision was made after skin disinfection to expose the surgical field. After the bregma and lambda were adjusted to the same horizontal level, two bone windows (one on each side) were drilled on the skull over the target regions following the coordinates derived from the rat brain atlas of Paxinos and Watson (2013). Then 0.1 μL of 10% biotinylated dextran amine (BDA, 10,000 MW, ThermoFisher Scientific, Waltham, MA, United States) or 4% Fluro-Gold (FG, Fluorochrome Inc., Denver, CO, United States) was pressure injected into the target brain regions [anteromedial nucleus (AM), A30 and A23~] of one hemisphere using a 0.5-μl Hamilton syringe. The following are the coordinates for tracer injections in the AM (AP -1.40, ML 0.90, DV 6.95), in A30 (AP -4.50, ML 1.00, DV 1.60 or AP -6.80, ML 0.70, DV 1.60), and in 23 ~ (AP -5.00, ML 1.85, DV 1.70 or AP -6.80, ML 1.58, DV 1.60). The needle was hold for 10 min after the tracer injection and then pulled out slowly. Finally, the incisions were sutured, and the rats were returned to their home cages after recovery on the warm bed.

### Brain processing and evaluation

7–10 days after the surgeries, the rats were deep anesthetized and perfused with 0.9% saline and 4% paraformaldehyde (PFA) in chilled 0.05 M phosphate buffer (PB, pH 7.3) in sequence. Then the brains were removed and immersed in the 4% PFA at 4°C overnight, and then cryoprotected in the PB containing 15 and 30% sucrose in succession for 3–4 days until the brains sank to the bottom. Each brain was separated into two halves with a midline cut. And each hemisphere was cut into 40 μm-thick coronal sections using a freezing microtome. The injection sites were evaluated by comparing them with the brain atlas (Paxinos and Watson, 2013). If the injection sites extend >80 μm beyond the defined borders of the ROI (e.g., A30 and A23~) the cases were excluded from further analysis. The borders of A30 and A23 ~ were determined by chemical markers and axon terminal zone of the AM afferents (see Results section).

### Histochemistry for BDA tracing

The procedure for BDA histochemistry has been described in our previous study (Lu et al., 2020; Chen et al., 2021). In brief, the sections derived from the hemisphere with BDA injections were thoroughly rinsed (at least three times 10 min each) in 0.05 M PB. Then the sections were incubated in 0.3% Triton X-100 in 0.05 M PB for 1 h and in Streptavidin-Biotin Complex solution (SABC kit, Boster Biological Technology) for 3 h at room temperature in sequence. After rinse in 0.05 M PB for three times, the sections were visualized with 0.05 M PB containing 0.05% 3, 3-diaminobenzidine (DAB). Finally, the sections were mounted on chrome alum and gelatin-coated glass slides, dehydrated in gradient alcohol and xylene, and finally coverslipped.

### Immunohistochemistry for crystallin

Two rats were used for IHC for Crym (crystallin, mu) to reveal expression pattern of Crym in A29, A30 and A23 ~ .The rats were perfused with 4% PFA and processed as described above. Sequential coronal sections containing A29, A30 and A23 ~ were immuno-stained using a method we described previously (Chen et al., 2021). Briefly, after rinses in 0.1 M of PB, the sections were incubated in 3% hydrogen peroxide solution for 10 min and in 5% bovine serum albumin (BSA) for 60 min for blocking. Next, sections were incubated at 4°C overnight with a solution containing 0.3% triton X-100 and the primary antibody [rabbit anti-Crym (PA5-65072, 1:1,000, Thermo Fisher Scientific, United States)]. Then, the sections were incubated with the secondary antibody solution (biotinylated goat anti-mouse/rabbit IgG, Boster Biological Technology, United States) followed by the Streptavidin-Biotin Complex solution (SABC kit, Boster Biological Technology) for 60 min each. After thorough rinses, the sections were visualized by incubating in 0.1 M of PB containing 0.05% 3, 3′-diaminobenzidine (DAB) and 0.01% hydrogen peroxide. Finally, the sections were mounted on chrome alum and gelatin-coated slides, dehydrated in gradient alcohol and xylene, and coverslipped.

### Image acquisition and processing

The images from the sections of the brains with FG injections were obtained using an epifluorescent microscope (Leica DM6B). The images from BDA-stained and Crym immune-stained sections were acquired with a scanner (Aperio CS2, Leica). All the selected images were processed with Adobe photoshop for image cropping, brightness and contrast adjustment, image composing, and structural annotation.

### Cell counts and statistics of labeled neurons

To compare the numbers of FG or BDA retrogradely labeled neurons in the AD, AV, and AM after A23 ~ and A30 injections in the rats, the numbers of these neurons in the three structures were counted on sequential sections containing these structures. These sections were grouped into five A-P levels and each level includes two closely adjacent sections. The following are the AP ranges for levels 1–5: level 1, B-1.32 to −1.44; level 2, B-1.52 to −1.64; level 3, B-1.72 to −1.84; level 4, B-1.92 to −2.04; level 5, B-2.04 to −2.16. Cell counts were performed using Image J and the statistics were done using one-way ANOVA and Tukey test (for each of the two pairs) or two-tailed unpaired *t*-test (for comparing cell numbers in the AM, AD or AV between A30 and A23 injections).

Since the main aims of this study is to distinguish A23 ~ from adjoining A30 and V2M and to reveal the brain regions with obvious connections with A23 ~ in the rats and mice, we mainly rely on large difference in connectivity, which is clearly visible with naked eyes. Thus, quantitative analysis of the density of labeled neurons and axon terminals was not carried out except for the labeled neurons in the anterodorsal (AD), anteroventral (AV) and anteromedial (AM) nuclei of the thalamus, which serves as an example to confirm the connections from the AD/AV and AM to A29 and A23~, respectively.

### Monkey brain data

Raw *in situ* hybridization (ISH)-stained sections for the gene *Enc1* and closely adjacent Nissl-stained sections were downloaded from the Allen Institute NHP Atlas data portal.^1^ Detailed description about the ISH data is openly available.^2^ The present study used the *Enc1*-ISH data and Nissl-stained images derived from 48 months-old Macaca monkey (*Macaca mulatta*; three cases, all male; experiment IDs: 100140502, 100144961 and 100148087).

### Mouse brain data

Raw ISH and connectivity data as well as related Nissl-stained sections were downloaded from Allen Institute portals^3^ and analyzed by the authors.^4^ The ISH data used in this study include ISH-stained sections for genes *Ddit4l, Npnt, Fam3c, Vamp1* and *Crym* (see Results section). These genes were selected using the gene selecting tool (AGEA; see text foot note 3).

The connectivity data used in this study include the cases/experiments with the anterograde viral injections almost entirely or largely restricted in the anteroventral nucleus (AV; 4 cases), or AM (6 cases), or A23 ~ (4 cases). The boundaries of these regions were determined according the brain atlas, molecular markers and unique afferents (see below). Based on these boundaries, we found 4 cases with the injections in the AV (experimental IDs: 614435699, 146046430, 292478008 and 286553311), 6 cases with the injections in the AM (experimental IDs: 146658170, 158840459, 266174045, 506947040, 514333422 and 592698832), and 4 cases with the injections in A23 ~ (experimental IDs: 593018150, 576341623, 575782182 and 562674923). Some representative cases are shown in the Results section with their IDs indicated. Other cases with obvious involvement in the adjoining regions were excluded from further analysis.

Additionally, four cases with the retrograde viral tracer (CAV-cre) injections in A23 ~ were also examined to confirm the major projections from the AM to A23 ~ in mice (experimental IDs: 521955016, 560,029,094, 479,115,470 and 501,787,135).

### Boundary determination of A23~, A30 and other related regions

The borders of A23 ~ and A30 as well as other related regions were determined based on the commonly used atlases [e.g., Paxinos and Watson (2013)], differential gene expression patterns or chemical markers (see Results section) and unique afferent projections (e.g., stronger AM afferents to A23 but not to A30; see Results section).

## Results

### Cytoarchitecture and topography of A30 and A23 in primates and rodents

**Figure 1:**
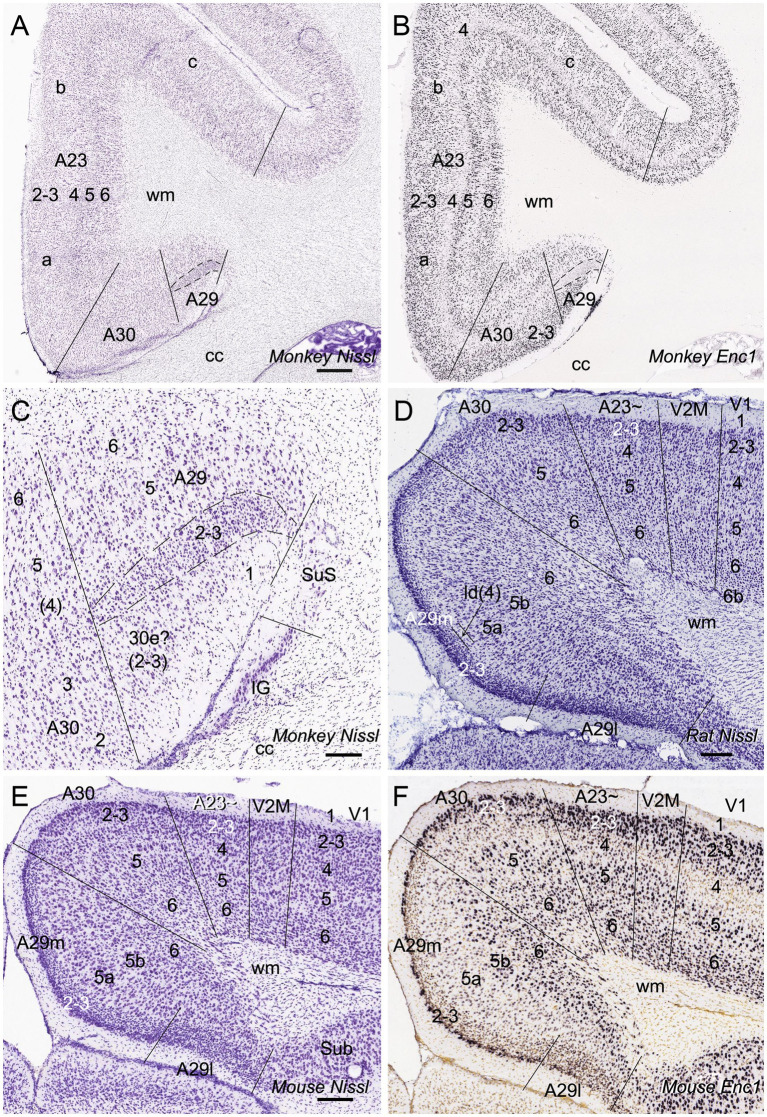
Cytoarchitecture and topography of A30 and A23 in the monkey and rodent. For all panels, Arabic numbers (1–6) indicate cortical layers while solid straight lines mark the regional borders. For orientation of each section, medial is at the left and dorsal is at the top. Species and stains are indicated at the bottom of each panel. Nissl and *Enc1*-ISH Data are derived from the Allen Non-human Primates Atlas (for monkey; https://www.blueprintnhpatlas.org/) and the Allen Mouse Brain Atlas (for mouse; https://mouse.brain-map.org/). **(A,B)** Locations, layers, subdivisions, and topography of areas 29, 30 and 23 (A29, A30 and A23) in the monkey. Two closely adjacent sections were stained for Nissl substances **(A)** and *Enc1-*ISH **(B)**. **(C)** Higher magnification view of A29 and A30 from panel **(A)**. Layers 2–3 (L2–3) of A29 is outlined by dashed lines and are densely packed with small granular cells. **(D)** Locations, layers, subdivisions, and topography of A29, A30 and A23 in the rat. The arrow indicates a cell-sparse zone (ld, lamina dissecans), which separates L2–3 and L5. **(E,F)** Locations, layers, subdivisions, and topography of A29, A30, and A23 in the mouse, showing on two closely adjacent sections stained for Nissl **(E)** and *Enc1*-ISH **(F)**. Note that L2 and L3 of A29 is distinguishable from each other and no extra layer appears superficial to L2–3 of A29. Also note the existence of a thick extra sublayer of L5 (L5a) in the mouse A29 with faint *Enc1* expression **(F)** although strong *Enc1* expression is observed in typical L5 of A29, A30 and A23 (L5b; **F**) as in the monkey **(B)**. Bars: 790 μm in **(A)** for **(A,B)**; 197 μm in **(C)**; 184 μm in **(D)**; 210 μm in **(E)** for **(E,F)**.

Human A23 has similar cytoarchitecture and topography to monkey A23 (Vogt et al., 2001). In monkeys, A23 is typically located dorsal to A30 while A29 adjoins A30 ventrally (e.g., Figures 1A,B). A29 is characterized by densely packed small granular cells in its superficial layers 2 and 3 (i.e., outer granular cells), which are not distinguishable from each other (outlined in Figure 1C). The thick deep layers (L5-6) of A29 are mainly composed of larger pyramidal cells (Figure 1C). It is noted that a layer of larger cells is observed superficial to the small-celled L2-3 of A29 and is termed A30e (30e in Figures 1C,E for extension) since A30e appears to be a lateral extension of L2-3 of A30 compared to rodent A29 (Figures 1D–F). Both A30 and A23 of the monkeys do not contain the densely packed outer granular cells, but A23 has a clear inner granular layer (L4) with no or faint *Enc1* expression. Both A30 and A23 have a thick L5 and an even thicker L6 with strong *Enc1* expression (Figures 1A,B).

Like in the monkeys, L2-3 of A29 in the rat (Figure 1D) and mouse (Figure 1E) also consist of densely packed outer granular cells (L2-3). However, L2-3 of A29 in the rodents are distinguishable from each other. L3 is densely packed with small cells and negative for *Enc1* while L2 is darkly stained and even more densely packed and is positive for *Enc1* (Figures 1D–F). Unlike in the monkeys, no extra A30e appears superficial to L2-3 of A29 in the rodents (Figures 1D–F). Interestingly, A29 appears to have an extra sublayer of L5 (L5a) in the rodents, which is thick and negative for *Enc1* compared to typical L5 (L5b), which is continuous with L5 in A30, A23 and neocortex, and positive for *Enc1* (Figures 1D–F). Both L5a and L5b of A29 in the mice express pan-L5 marker genes such as *Etv1, Rbp4* and *Adcyap1* (see Hu et al., 2020). A29 in the rodents displays a thinner L6 compared to the monkeys. Topographically, A30 in the rodents is located dorsolateral to A29 while A23 ~ adjoins A30 laterally (Figures 1D,E). A30 in the rodents has less densely packed L2-3, which are *Enc1* positive and not distinguishable from each other, and thick L5-6 with positive *Enc1* (Figures 1D–F). A23 ~ in the rodents has slightly thicker L2-3 and slightly thinner L5-6 compared to A30 as well as larger cells in L6 compared to adjoining V2M. It is obvious on coronal sections that A23 in the primates is much wider/larger in size than A29 (e.g., Figure 1A) while A23 ~ in the rodents is much narrower/smaller in size than A29 although the relative size of A30 appears comparable in both primates and rodents.

### Differential gene expression of A30 and A23 ~ in rodents

**Figure 2:**
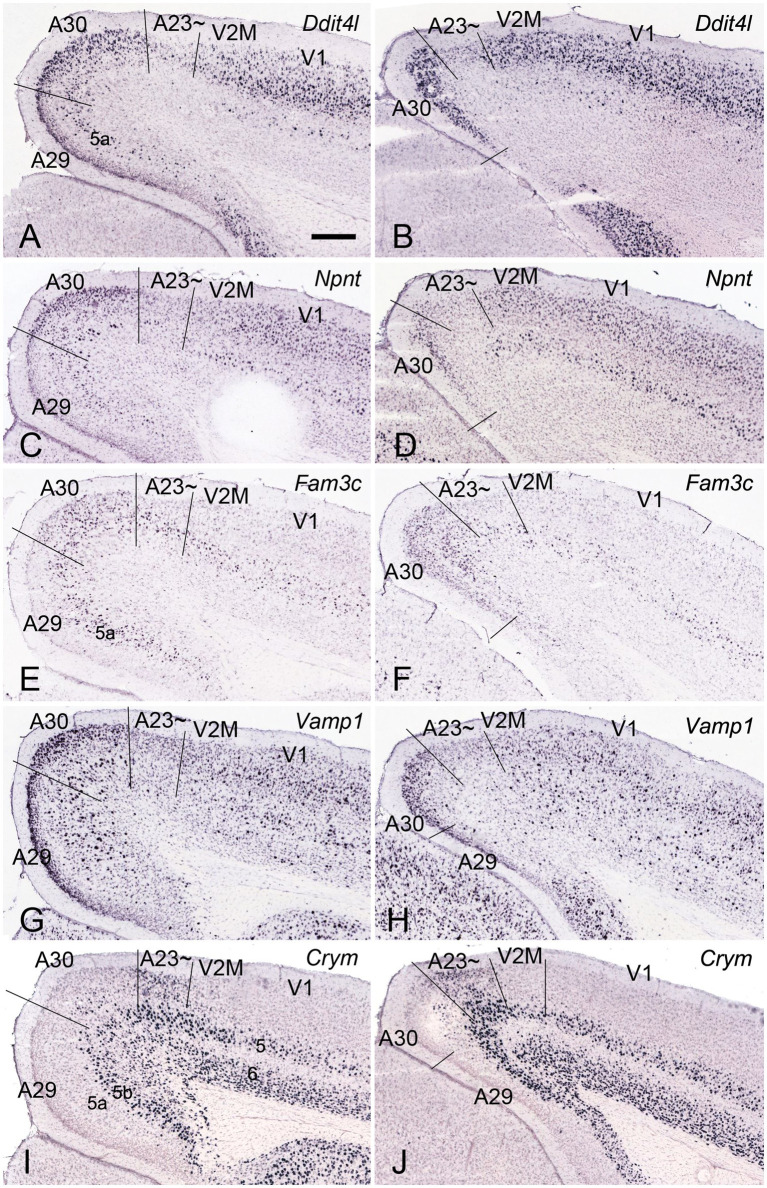
Differential gene expression in mouse A30 and A23~. **(A–H)** expression of *Ddit4l* **(A,B)**, *Npnt* **(C,D)**, *Fam3c* **(E,F)** and *Vamp1* **(G,H)** in L2–3 of A30 but not A23~. **(I,J)** *Crym* expression in L2–3 of A23 ~ but not A29 and A30. Note that L5a of A29 is negative for *Crym*. Left and right columns show the regions at the anterior and posterior levels, respectively. Bars: 300 μm in **(A)** for all panels.

In general, it is difficult to differentiate A30 from A23 ~ in the rodents based on Nissl staining. However, regional gene expression patterns could be helpful in distinguishing adjacent regions (e.g., Chen et al., 2022). To show the difference between A30 and its lateral region (A23~) as well as the borders of these two regions along the anterior–posterior (A–P) axis, we searched the Allen Mouse Brain Atlas (https://mouse.brain-map.org) for differential gene expression. We find that many genes such as *Ddit4l, Npnt, Fam3c and Vamp1* are strongly expressed in L2-3 of A30 (RSag) while some other genes such as *Crym* and Col*6a1* are expressed in L2-3 of A23 ~ (RSagl) but not of A29 (RSg) and A30 (Figure 2), suggesting that the RSagl (A23~) is different from RSag (A30). Using these gene markers, the boundaries of A30 and A23 ~ can also be clearly identified (Figure 2). Since RSagl (A23~) is located lateral to RSag (A30) and, in primates, the region dorsal/lateral to A30 is conceptualized as PCC (mainly A23), it is likely that the RSagl region corresponds roughly to A23 ~ and thus it is better to treat it as possible A23 ~ rather than as a part of RSag (A30).

**Figure 3:**
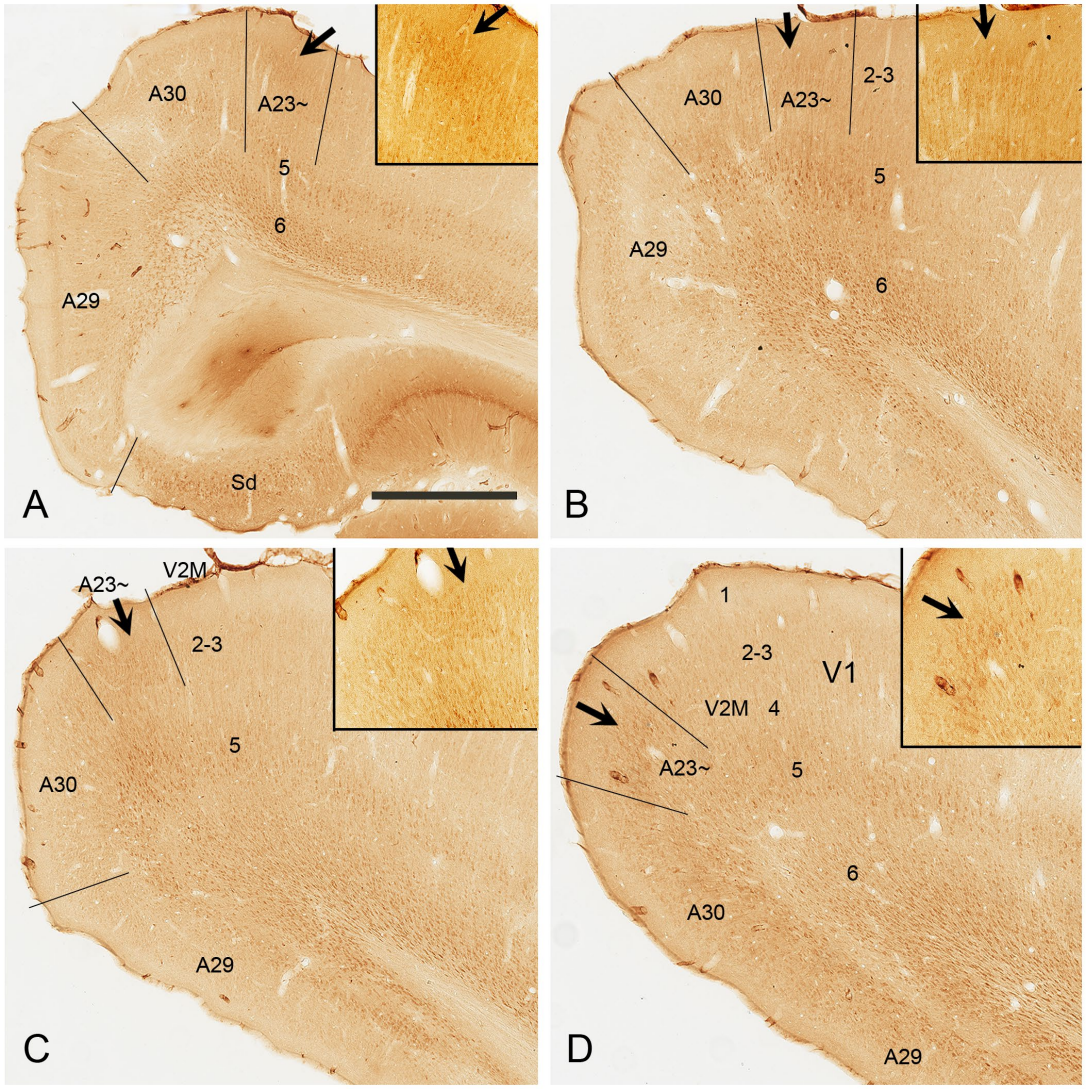
Crym expression in A29, A30 and A23 ~ of the rat revealed with immunocytochemistry. **(A–D)** Sequential coronal sections from the anterior **(A)** to posterior **(D)** levels showing the Crym staining pattern in A23 ~ and adjoining regions. Crym-stained neruons are mainly seen in the deep layers (L5 and L6) of A29, A30, A23 ~ and the visual cortices (V2M and V1). Relatively weaker Crym labeling is observed in L2–3 of A23 ~ with faint or no labeling in other adjoining regions. (Insets in **A–D**) Higher magnification views of L2–3 in A23~, showing the weaker Crym labeling in L2–3. The arrows in **(A–D)** and in the insets point to the corresponding regions. Bar: 600 μm in **(A)** for panels **(A–D)**; 300 μm in the Inset in **(A)** for all Insets.

Immuno-stained sequential sections from the rat brains show a similar pattern of Crym expression to that of mouse revealed with *Crym*-ISH. Generally, Crym-stained neurons in the rats are mainly seen in layers 5 and 6 of all the cortical regions adjoining A23~. However, weaker but clear Crym labeling is only observed in layers 2–3 of A23 ~ with faint or no labeling in other regions (Figure 3 and the insets). This pattern is helpful for determining the borders of A23 ~ in the rats.

### A23 ~ but not A30 receives major inputs from anteromedial thalamic nucleus in rats

**Figure 4:**
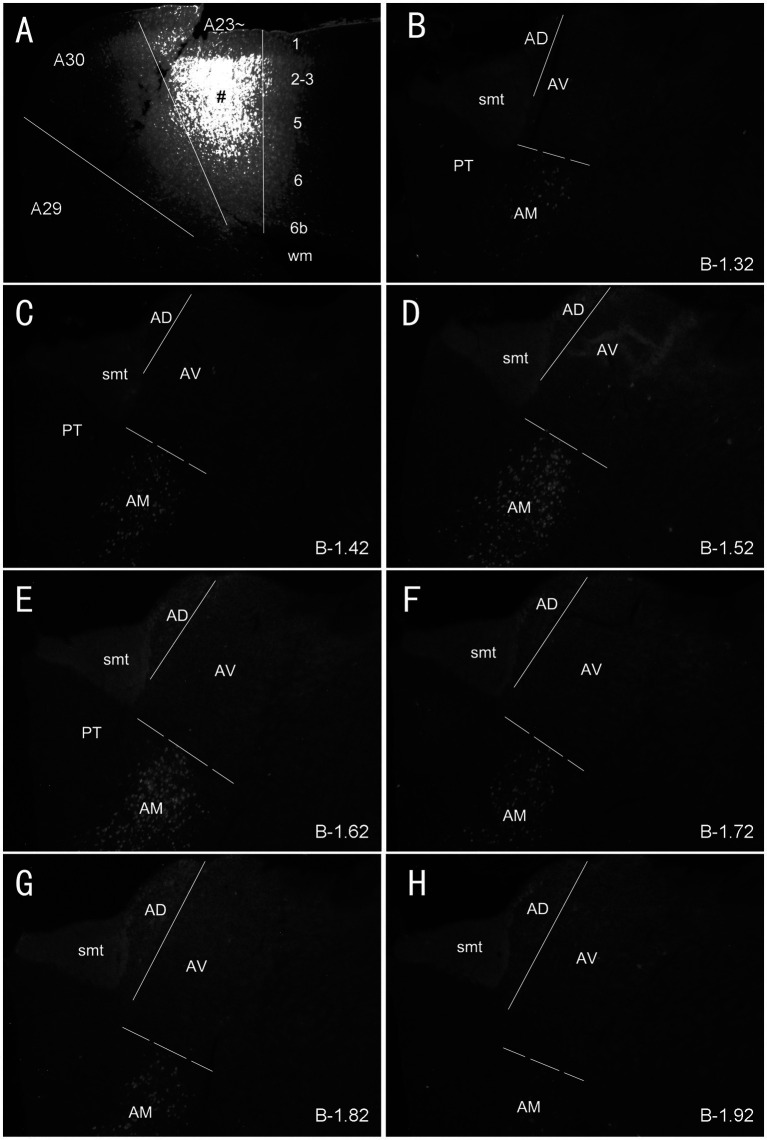
Labeled neurons in the AM following FG injection in rat A23~. **(A)** One FG injection site (#) in A23~. **(B–H)** Sequential coronal sections from the anterior **(B)** to posterior **(H)** levels showing FG labeled neurons in the AM. The proximate AP coordinate for each level was indicated at the bottom of each panel. The borders between the AD and AV and between AV and AM are indicated by a solid line and a dashed line, respectively. Bar: 500 μm in **(A)** for all panels.

**Figure 5:**
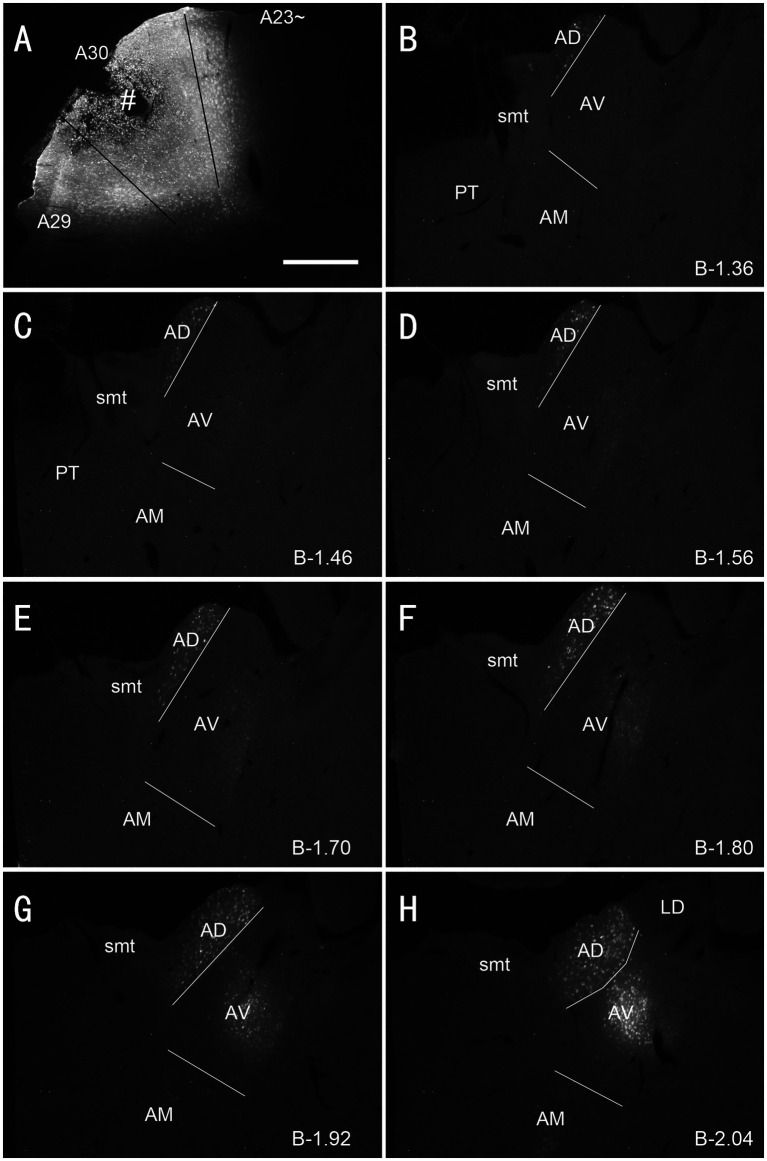
Labeled neurons in the AD and AV following FG injection in rat A30. **(A)**. One FG injection site (#) in A30 with slight involvement in A29. **(B–H)** Sequential coronal sections showing FG labeled neurons mostly in the AD and AV with few in AM. The proximate AP coordinate for each level was indicated at the bottom of each panel. Bar: 500 μm in **(A)** for all panels.

As mentioned above, the monkey AM projects to A23 while the AV and AD projects to A29 and A30 (Baleydier and Mauguiere, 1980; Vogt et al., 1987). To determine whether A23 ~ receives major inputs from the AM in the rats we injected the retrograde tracer Fluoro-Gold (FG) in the region corresponding to A23 ~ described above. After successful FG injections (4 cases), many retrogradely labeled neurons were found in the AM with no or few in AD and AV. For example, following one single FG injection in A23 ~ (Figure 4A) the labeled neurons are distributed across the A–P extent of the AM with no or few in other thalamic regions including the AD, AV and PT (Figures 4B–H). Some labeled cells were also observed in the ventromedial thalamic nucleus (VM) underneath the posterior AM. In contrast, when the FG injections were placed in A30 (4 cases with 2 cases slightly involved in adjoining A29 but not A23~), FG labeled neurons are mostly seen in the AD and AV with no or few labeled neurons in the AM (e.g., Figures 5A–H). These results indicate that both the AD and AV but not the AM project to A30. These findings are consistent with those in the monkeys (Vogt et al., 1987).

**Figure 6:**
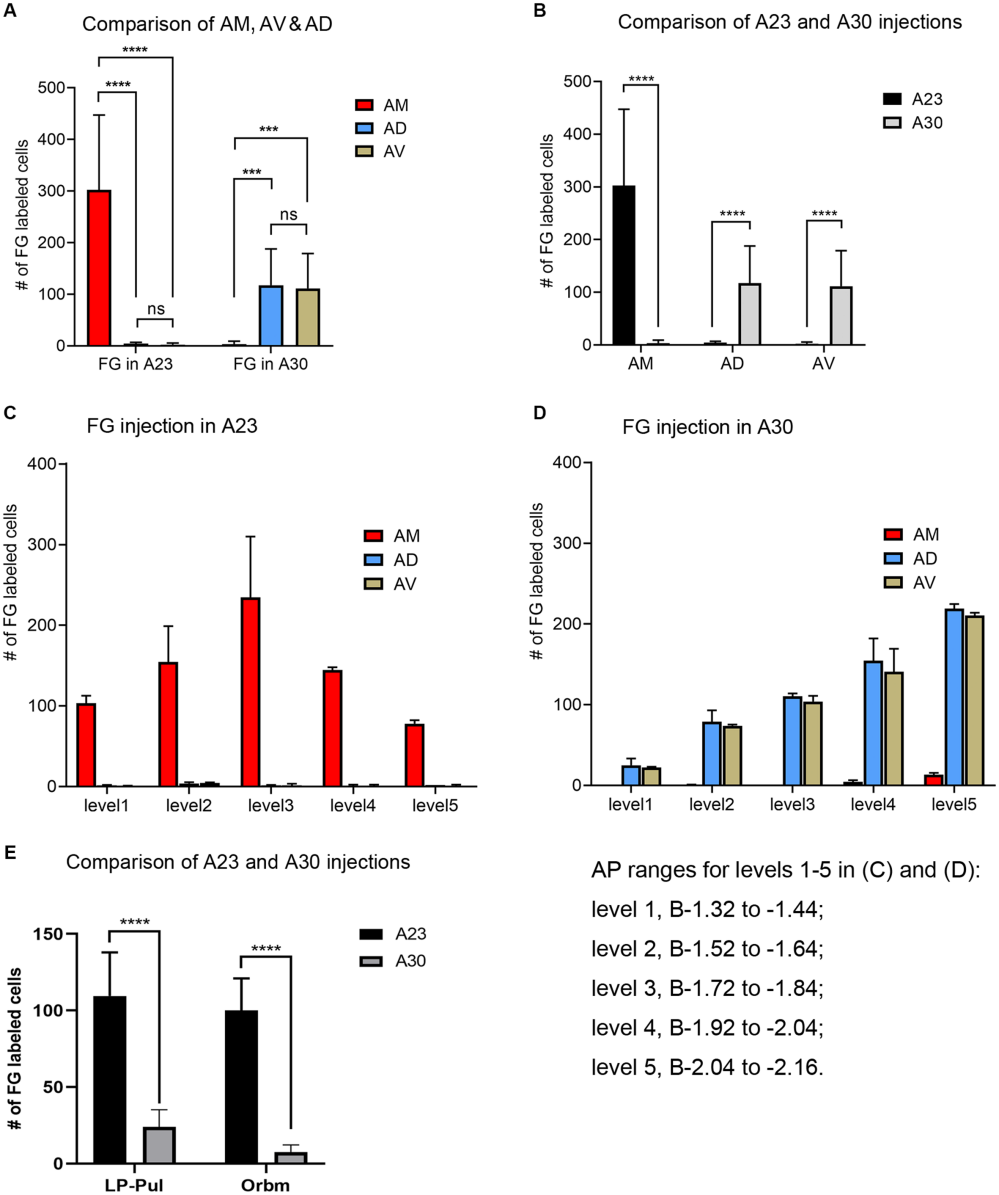
Quantitative analysis of FG labeled neurons in the AD, AV, AM, LP-Pul and ORBm of the rats. **(A)** Comparison of the cell counts in the AM, AD and AV following A23 ~ (left) and A30 (right) FG injections. ***p < 0.001; ****p < 0.0001. **(B)** Comparison of the cell counts in each of the anterior thalamic nucleus between A23 ~ and A30 injections. ****p < 0.0001. **(C)** Example of the cell counts along the A–P extent in one case with an FG injection in middle/posterior A23 ~ (mean ± SD). **(D)** Example of the cell counts along the A–P extent in one case with an FG injection in posterior A30 (mean ± SD). The AP ranges for level 1 to level 5 in **(C,D)** are same and indicated underneath panel **(D)**. **(E)** Comparison of the cell counts in the LP-Pul and ORBm resulted from A23 ~ and A30 injections. ****p < 0.0001.

**Figure 7:**
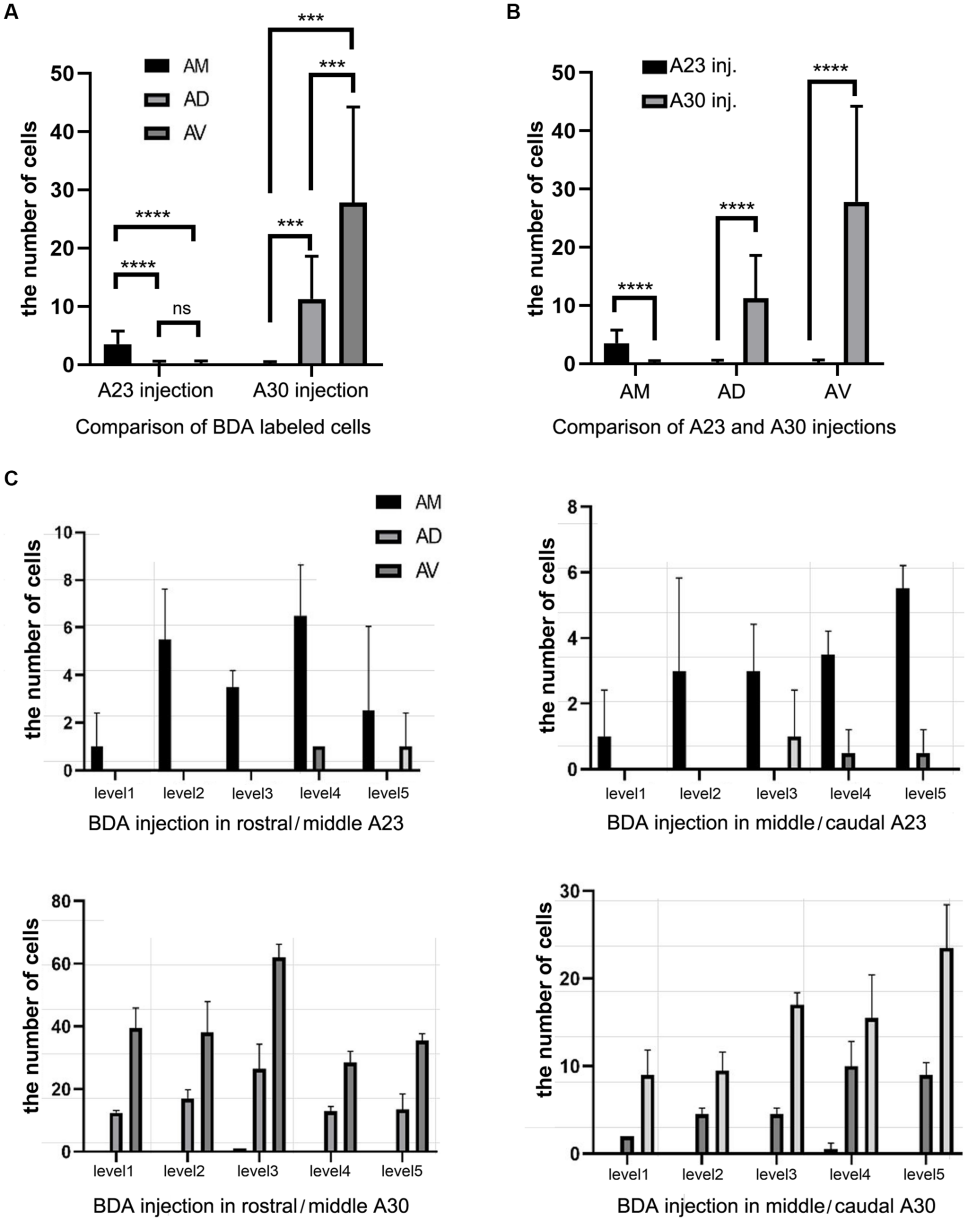
Quantitative analysis of BDA labeled neurons in the AD, AV, and AM of the rats. **(A)** Comparison of the cell counts in the AM, AD and AV following A23 ~ (left) and A30 (right) BDA injections. ***p < 0.001; ****p < 0.0001. **(B)** Comparison of the cell counts in each of the anterior thalamic nucleus following A23 ~ and A30 injections. ****p < 0.0001. **(C)** Examples of the cell counts along the A–P extent in four cases with BDA injections in A23 ~ (top) and A30 (bottom) (mean ± SD). The AP ranges for level 1 to level 5 are the same as in Figure 6D.

Quantitative analysis of the labeled neurons in the AD, AV and AM following FG injections in A23 ~ or A30 further indicates that A23 ~ but not A30 receives major inputs from the AM (Figure 6A, left) [F (2, 27) = 17.42, *p* < 0.0001, (R-squared: 0.7597)] whereas A30 but not A23 ~ receives inputs from both the AD and AV (Figure 5A, right) [F (2, 27) = 7.833, *p* < 0.001, (R-squared: 0.4885)]. Comparison of the labeled neurons resulted from FG injections in A23 ~ and A30 has further confirmed the differences (*p* < 0.0001; see Figure 6B). It is also noted that FG Injections in the middle/posterior A23 ~ lead to labeled neurons across the A-P extent of the AM with more in the middle levels (e.g., Figure 6C) while the injections in the posterior A30 produce labeled neurons mainly in the posterior AD and AV (e.g., Figure 6D). Similar results are found for biotinylated dextran amine (BDA, a bidirectional tracer) labeled neurons after BDA injections in A30 and A23 ~ (4 cases; Figure 7). Additionally, comparison of the labeled neurons in the lateroposterior nucleus-pulvinar complex (LP-Pul) and medial orbitofrontal cortex (ORBm) resulted from FG injections in A23 ~ and A30 has also showed significant differences (*p* < 0.0001; see Figure 6E).

**Figure 8:**
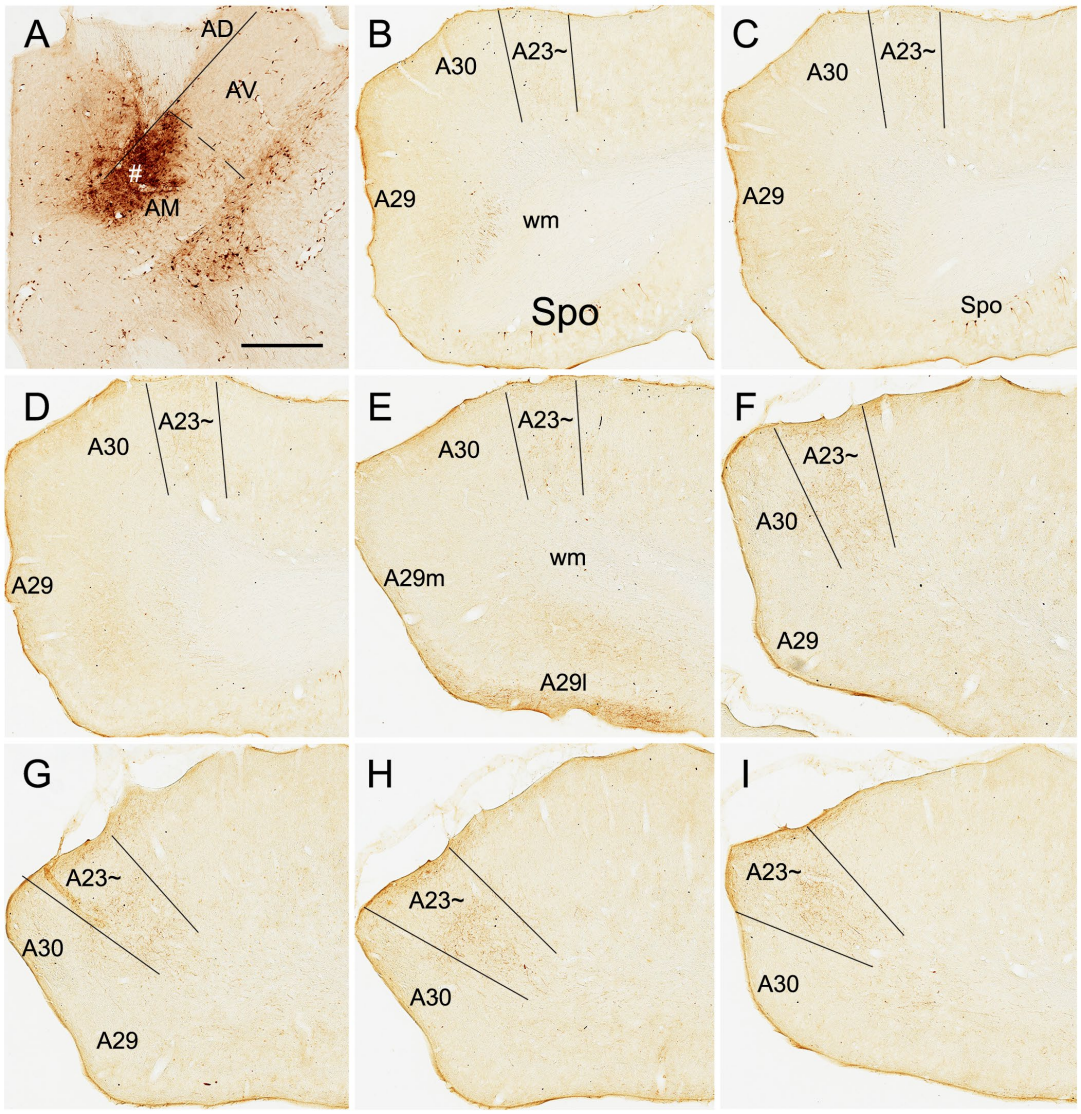
AM projections to A23 ~ after a small BDA injection in the rat AM. **(A)** The BDA injection site (#) in the mediodorsal part of the AM. **(B–I)** Sequential coronal sections from the anterior **(B)** to posterior **(I)** levels showing BDA labeled axon terminals in A23~, which are distributed more densely at the posterior levels **(F–I)**. Bar: 500 μm in **(A)** for all panels.

**Figure 9:**
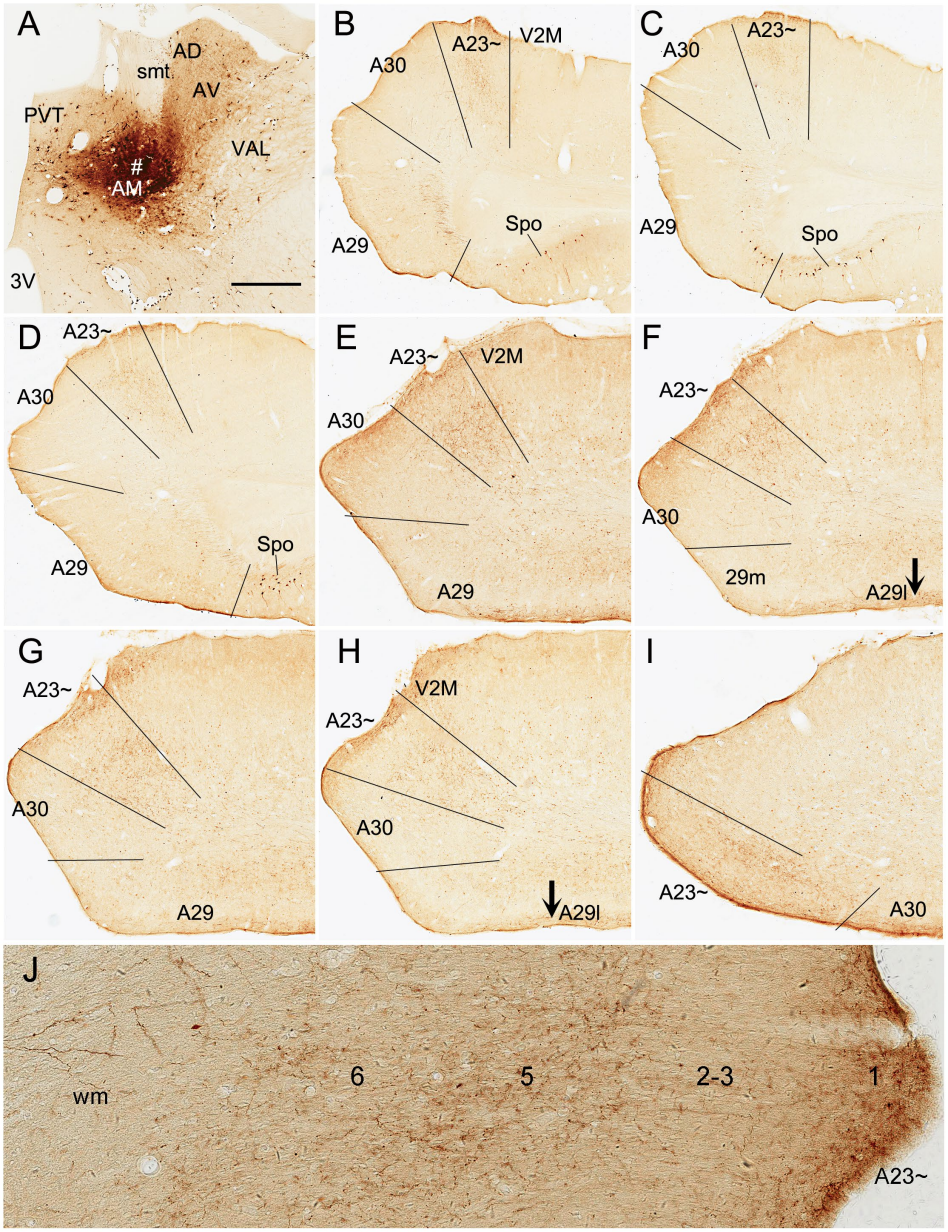
AM projections to A23 ~ after a larger BDA injection in the rat AM. **(A)** The BDA injection site (#) in the AM. **(B–I)** Sequential coronal sections from the anterior **(B)** to posterior **(I)** levels showing BDA labeled axon terminals in A23~. The regional borders are indicated by solid lines in each panel. Labeled terminals are mainly in L1 of A29l are indicated by the arrows in **(F,H)**. Note that some BDA labeled neurons are observed in the polymorphic layer of the subiculum (Spo in **B–D**). **(J)** Higher magnification view of the labeled axon terminals in A23 ~ shown in **(B)**. Bar: 500 μm in **(A)** for all panels.

Based on above findings it is reasonable to infer that anterograde tracer injections in the AM would produce obvious terminal labeling in A23 ~ rather than in adjoining A30 and V2M, and thus enable identification of the borders of A23~. Indeed, following the BDA injections into the AM of the rats (4 cases), BDA labeled axon terminals were observed across the A-P extent of A23~. For example, one small BDA injection in the dorsomedial part of the AM results in terminal labeling across the A-P extent of A23 ~ with densely and weakly labeled terminals in the anterior and posterior A23~, respectively (Figure 8). In contrast, following one larger BDA injection covering most of the AM (Figure 9A), the resulted terminal labeling is clearly observed in almost all A-P extent of A23 ~ (Figures 9B–I). Labeled terminals are distributed mainly in L1, L5 and L6 of A23 ~ (Figure 9J). The labeled axon terminals in L1 of A23 ~ extend slightly into A30 (medially) and the medial visual cortex (V2M; laterally) while those in L5-6 mostly concentrate in A23~. It is noted that L1 of the lateral A29 (A29l) also contains labeled terminals, but this labeling is far away from that in A23 ~ [separated by A30 and medial A29 (A29m)]. This labeling pattern enables the identification of the medial and lateral borders as well as the anterior and posterior borders of A23 ~ (Figures 9B–I). It is estimated that A23 ~ in the rats corresponds roughly to the RSagl defined by Swanson (2004) or medial portion of the V2M (V2MM) defined by Paxinos and Watson (2013). Specifically, rat A23 ~ anteriorly starts at the level above the CCS (the level slightly anterior to Figure 9B), and posteriorly ends at the level, where the terminals are located at the medial edge of the visual cortex (at the level of Figure 9I; about 8.70 mm posterior to the bregma based on the Paxinos atlas).

### Anteromedial thalamic inputs define A23 ~ in mice

**Figure 10:**
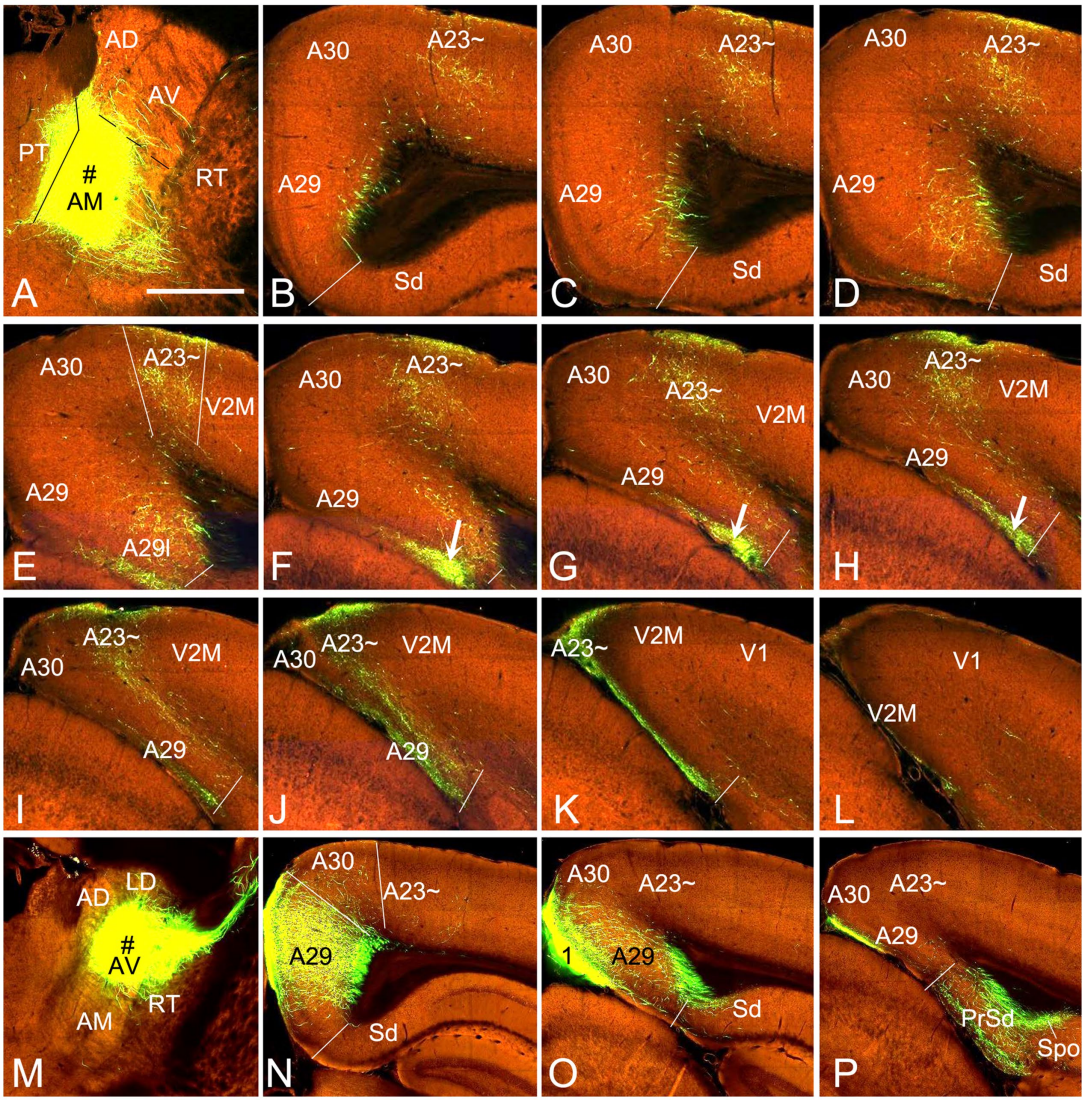
AM and AV projections to A23 ~ and A29 revealed with the viral tracers in the mouse. **(A)** One injection site (# in **A**; experiment ID: 266174045) in the AM with slight involvement in the paratenial nucleus (PT). **(B–L)** Sequential coronal sections from the anterior **(B)** to posterior **(L)** levels showing labeled axon terminals in A23 ~ and area 29 L (arrows in **F–H**) resulted from the AM injection. Like in the rat, labeled terminals are mainly detected in L1 and L5-6 of A23 ~ and L1 of A29l. **(M–P)** One injection site (# in **M**; experiment ID: 286553311) centered in the AV of the mouse results in labeled axon terminals in L1 and L5–6 of A29 with much fewer in A30 and none in A23 ~ **(N,O)**. Note that the AV (but not AM) injections produce axon terminal labeling in the polymorphic layer of the subiculum (Spo in **P**). Bar: 560 μm in **(A)** for all panels.

**Figure 11:**
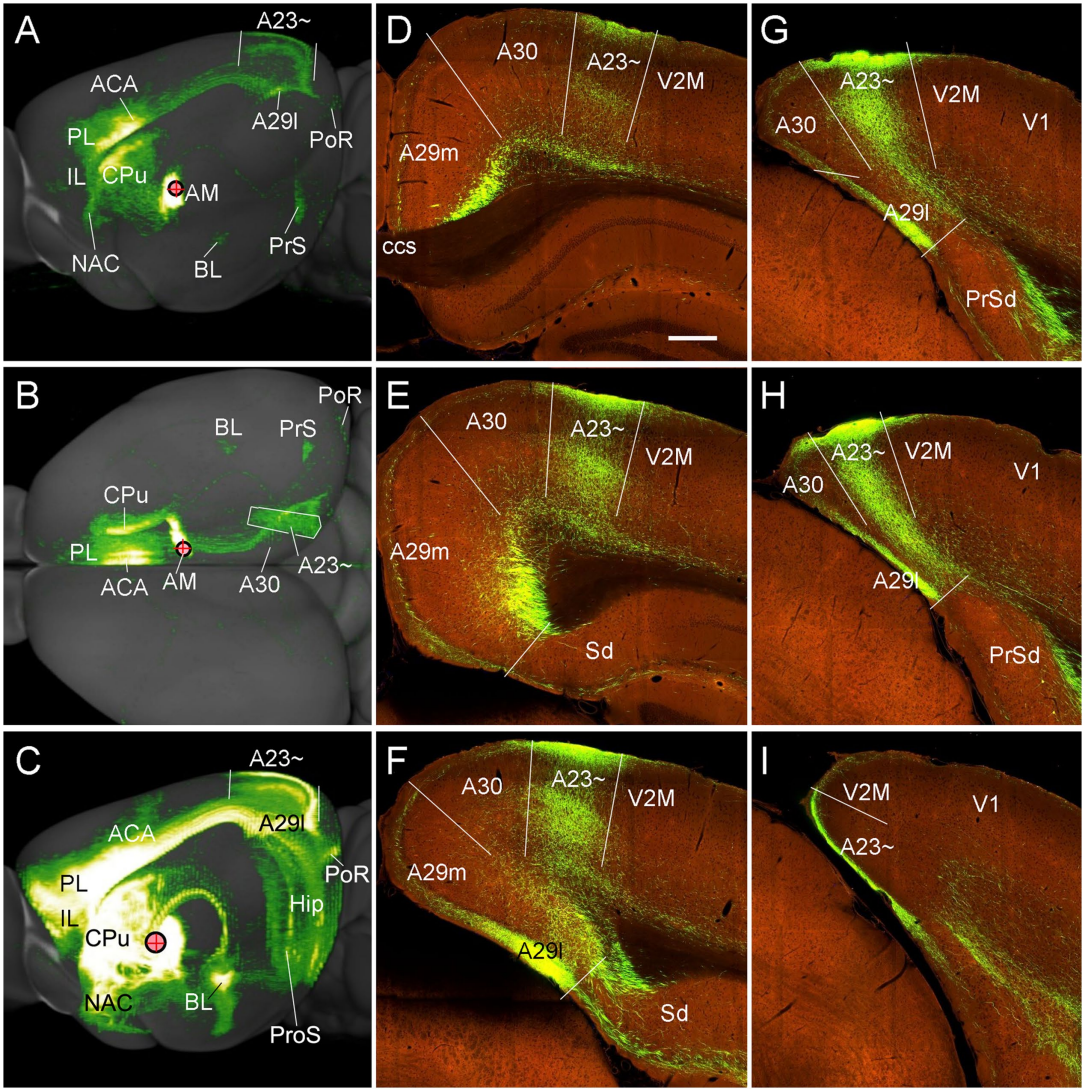
Major targets of the AM projections revealed with anterograde viral tracers in the mice. **(A,B)** Brain-wide AM projection pattern on the lateral **(A)** and dorsal **(B)** aspects of the brain. Individual A–P sections showing the labeled terminals are demonstrated in Figures 10A–L. It is obvious that the major targets of the AM projections include the ACA, PL, A23~, A29l, CPu and PrS. Note that the injection is slightly involved in the PT, which leads to some terminal labeling in the IL, NAC and BL (see Vertes and Hoover, 2008). **(C)** One large injection site covering entire AM and adjoining PT and Re (experiment ID: 159331462) results in strong terminal labeling in A23 ~ with few in A30. **(D–I)** Sequential coronal sections showing the A–P extent of A23 ~ revealed by the injection shown in **(C)**. The strongly labeled axon terminals in A23 ~ make this region easily identifiable. Generally, A23 ~ starts anteriorly at the level overlying the CCS **(D)** and ends posteriorly at the medial edge of the posterior cortex slightly posterior to the level H. Bar: 280 μm in **(D)** for panels **(D–I)**.

**Figure 12:**
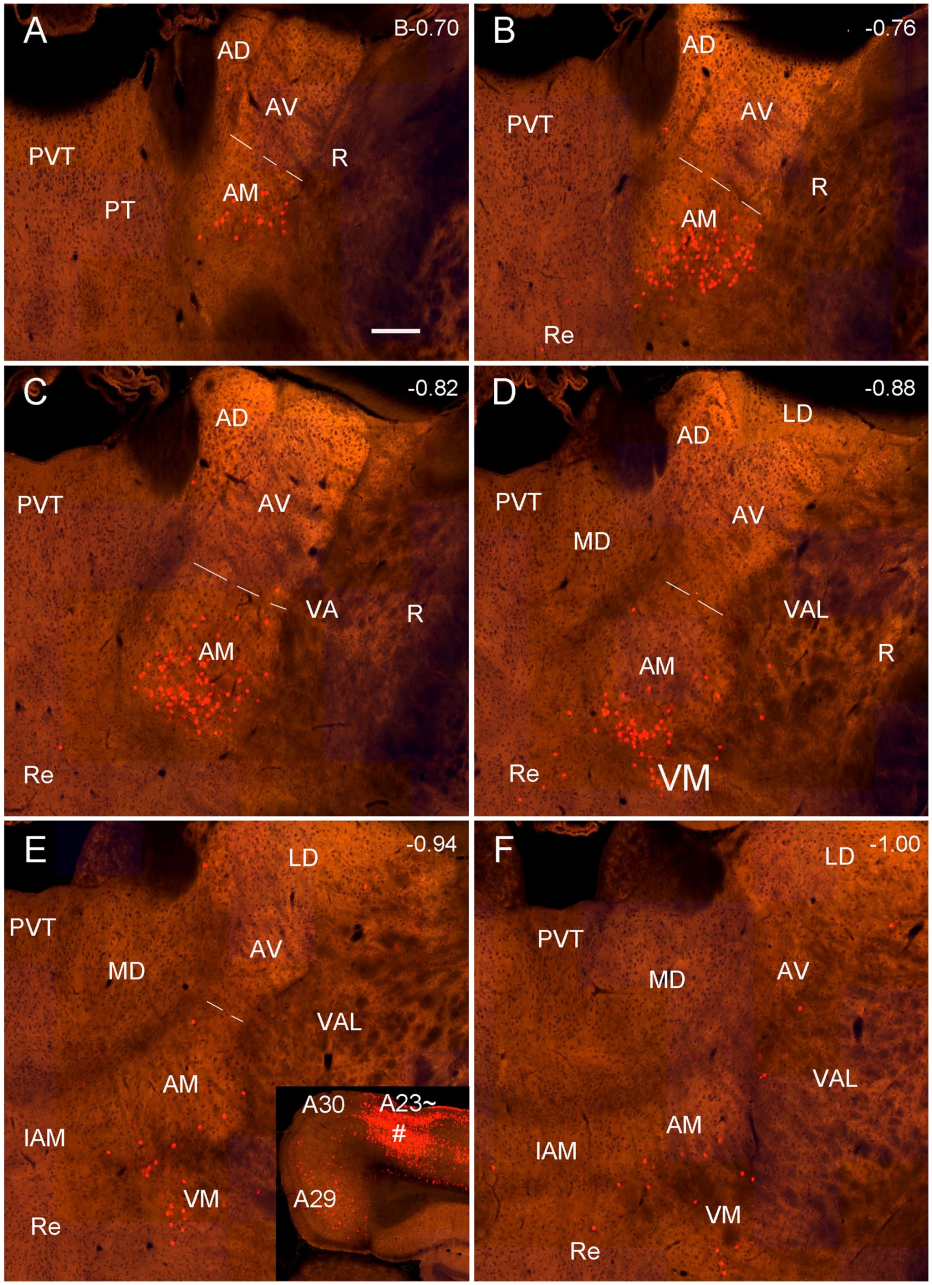
Labeled neurons in the AM revealed with the retrograde CAV2-Cre viral tracer injection in the mouse A23~. **(A–F)** Sequential coronal sections from the anterior **(A)** to posterior **(F)** levels showing CAV2-Cre labeled neurons in the AM, VM and A29. The inset in **(E)** shows the injection site (#) in the anterior A23 ~ (experiment ID: 521955016). Note that the labeled neurons are mainly seen in the ventral part of the AM. Bar: 200 μm in **(A)** for all panels.

To confirm above findings in mice, we performed a survey on the Allen Mouse Connectivity dataset (see text foot note 4), in which more sensitive anterograde viral tracers were used for the experiments (6 cases; experimental IDs: 146658170, 158840459, 266174045, 506947040, 514333422 and 592698832). As in the rats, the AM injections produced dense terminal labeling in A23 ~ rather than in A30 and V2M. For instance, one injection of the viral tracers into the AM of the mouse (Figure 10A) results in dense terminal labeling mainly in L1, L5 and L6 of A23 ~ with no or few in adjoining A30 and V2M (Figures 10B–L). The overall projection pattern from this case is shown in the lateral and dorsal aspects of the brain (Figures 11A,B). It is obvious that five major target regions of the AM projections are the anterior cingulate cortex [ACC, including the anterior cingulate area (ACA) and prelimbic area (PL)], caudate-putamen (CPu), presubiculum (PrS), A29l and A23 ~ (Figures 11A,B). It is also clear from the dorsal aspect view that A23 ~ is a band-like terminal zone (outlined in Figure 11B) located lateral to A30, which barely receives inputs from the AM. As a comparison, the viral tracer injections in the AV (e.g., Figure 10M) lead to heavy terminal labeling in L1 and moderate labeling in L3 and L5-6 of A29m with much less in A30 and no in A23 ~ (Figures 10N–P). These findings are further confirmed with the retrograde viral tracer (CAV2-Cre) injections in A23 ~ (Figure 12).

To determine and verify the full A-P extent of A23 ~ in the mice, we also examined some cases with even larger injections which cover entire A-P extent of the AM as well as the adjoining parataenial nucleus (PT) and reuniens nucleus (Re) of the thalamus (3 cases). For example, as shown in Figure 11C, although strong terminal labeling is found in many other structures the A-P extent of the labeled terminals in A23 ~ is comparable to that revealed in Figure 11A. Specifically, the most anterior level of A23 ~ (containing densely labeled terminals from the AM) is at the level above the CCS (Figure 11D) while the most posterior level is at the level between panels (H) and (I) of Figure 11 (about 4.36 mm posterior to the bregma according to the mouse brain atlas (Paxinos and Franklin, 2012). It is noted that the location of A23 ~ slightly shifts from lateral (Figure 11D) to medial (Figure 11H) positions along the A-P axis (Figures 11D–I). It should also be mentioned that the PT and Re do not project to A30 and A23 ~ (Vertes and Hoover, 2008) although these two regions are involved in the large injection site. The PT mainly projects to the IL, NAC, and BL (Vertes and Hoover, 2008) while the Re mostly projects to the prosubiculum (ProS in Figure 7C; also see Figure S9 of Ding et al., 2020), CA1, piriform, insular, perirhinal (PRC) and prefrontal cortices (Vertes et al., 2006).

### Afferent and efferent connections of A23 ~ in the rats

**Figure 13:**
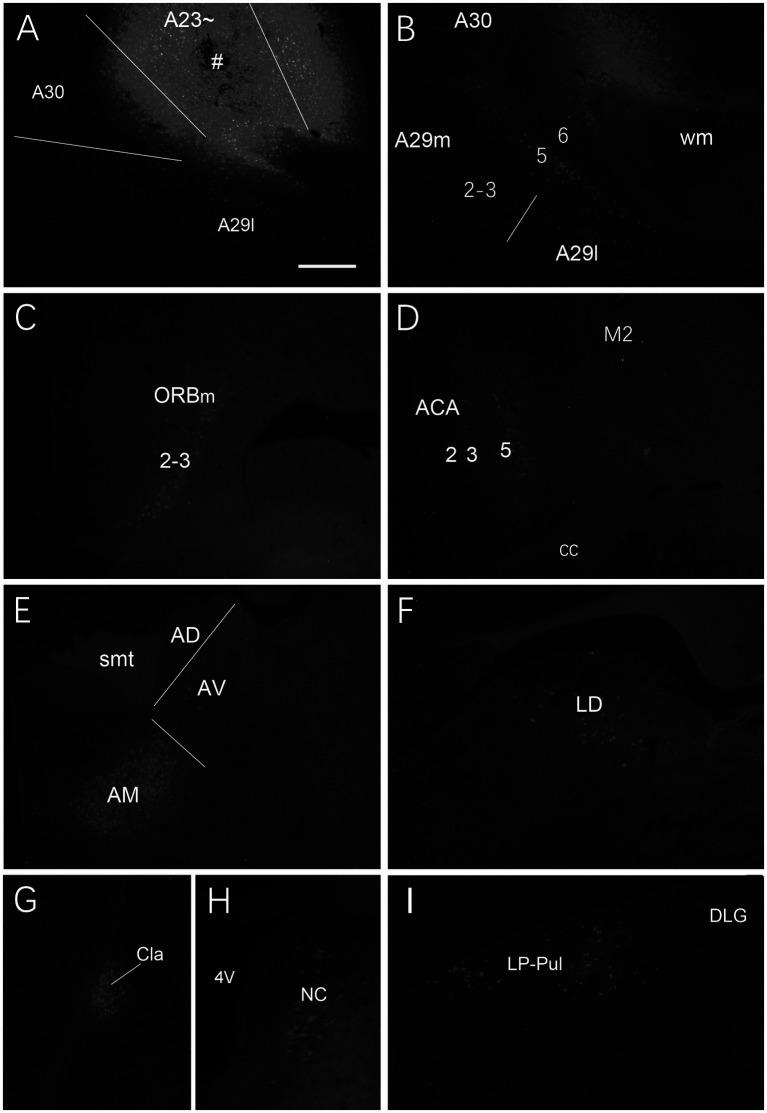
Afferent projections of A23 ~ revealed with FG in the rat. **(A)** One FG injection site (#) in A23~. **(B–H)** Examples of FG labeled neurons in A29 **(B)**, ORBm **(C)**, ACA and M2 **(D)**, AM **(E)**, LD **(F)**, Cla **(G)**, nucleus coeruleus (NC; **H**) and medial LP-Pul **(I)**. Bar: 500 μm in **(A)** for all panels.

**Figure 14:**
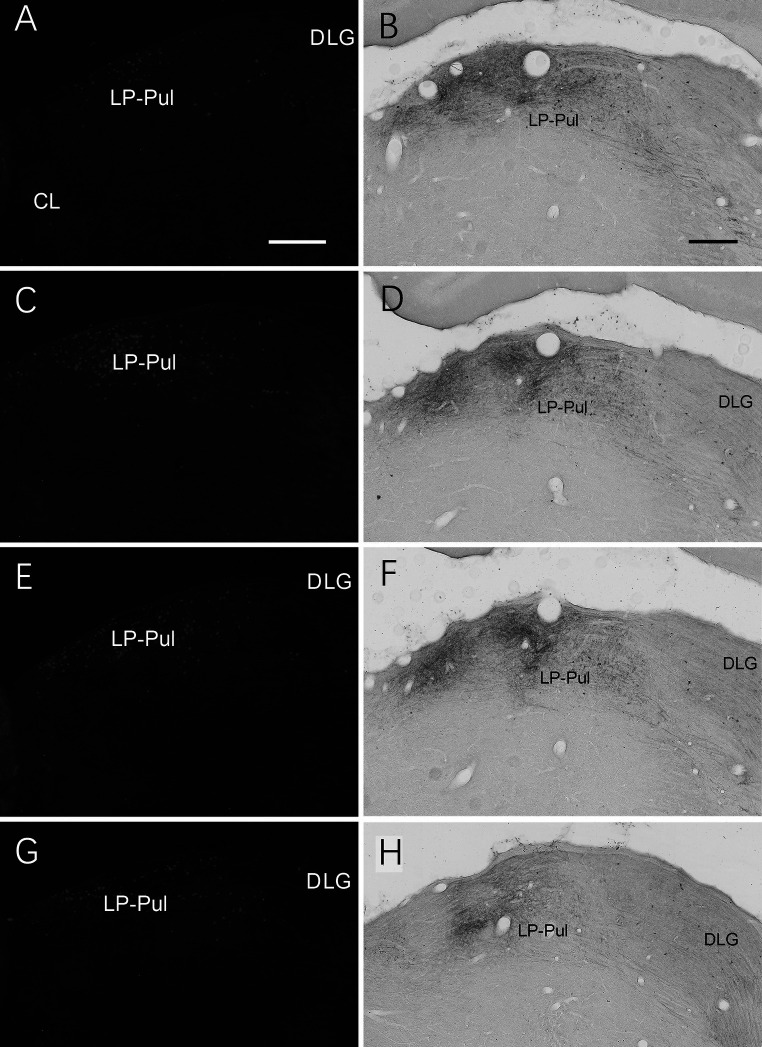
Labeled neurons and axon terminals in the LP-Pul of the rats. **(A,C,E,G)** Sequential coronal sections from the anterior **(A)** to posterior **(G)** levels of the LP-Pul showing the labeled neurons in the medial part of the LP-Pul after FG injection in A23~. **(B,D,F,H)** Sequential coronal sections from the anterior **(B)** to posterior **(H)** levels of the LP-Pul showing the labeled axon terminals in the medial LP-Pul after the BDA injection in A23~. Bars: 500 μm in **(A)** for panels **(A,C,E,G)**; 300 μm in **(B)** for panels **(B,D,F,H)**.

We also investigated brain-wide distribution of the retrogradely labeled neurons following FG injections in A23 ~ of the rats (4 cases). For instance, as shown in Figure 13, after one FG injection into A23 ~ (Figure 13A), retrogradely labeled neurons are seen in cortical A29 (in L3 and L5; Figure 13B), ORBm (in L2-3; Figure 13C), ACA (in L3 and L5; Figure 13D) and parietal association cortex (PtA). Sparsely labeled neurons are also detected in L5 of the secondary motor cortex (M2; Figure 13D), PL, postrhinal cortex (PoR), medial entorhinal cortex (MEC), a part of lateral secondary visual cortex (V2L) and the posterior part of the temporal association cortex (TeA) located posterior to the primary auditory cortex. The subcortical regions containing the labeled neurons include the AM (Figure 13E), laterodorsal thalamic nucleus (LD; Figure 13F), claustrum (Cla; Figure 13G), nucleus coeruleus (NC; Figure 13H), forebrain basal nucleus (not shown) and the medial part of the LP-Pul (Figure 13I). The LP-Pul, corresponding to the LP and pulvinar (Pul) of the monkeys (Baleydier and Mauguiere, 1985), contains labeled neurons in its full A-P extent (Figures 14A,C,E,G).

**Figure 15:**
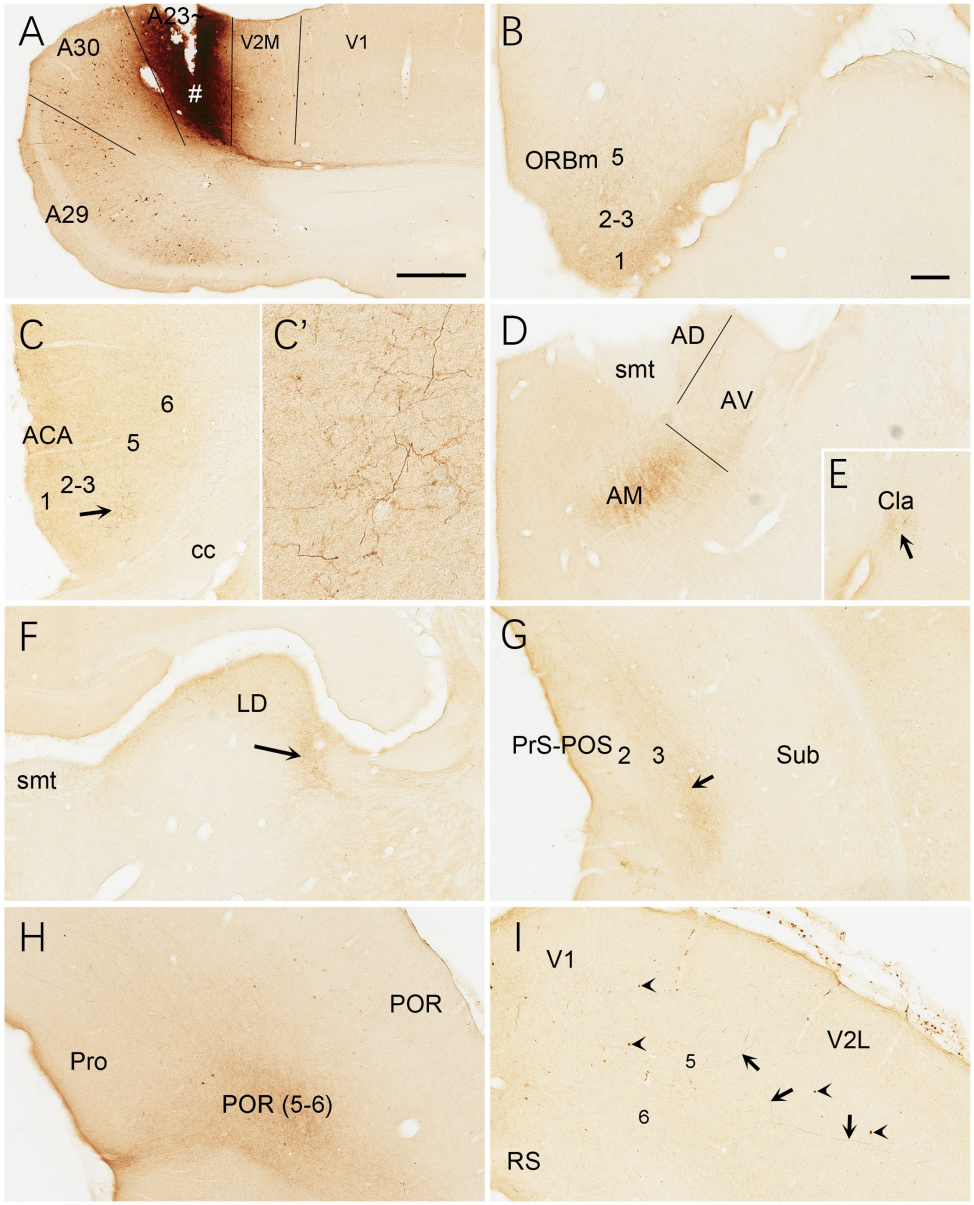
Efferent projections of A23 ~ revealed with BDA in the rat. **(A)** One BDA injection site (#) restricted in A23~. **(B–I)** BDA labeled axon terminals in L1 of the ORBm **(B)**, L1 and L3 of the ACA **(C,C′)**, AM **(D)**, Cla **(E)**, LD **(F)**, L3 of the PrS-PoS, L5–6 of the PoR **(H)** and V2L (arrows in **I**). The arrows in **(C,E,F,G)** point to the regions with dense axon terminals. A high magnification view of the terminals in the ACA (arrowed region) is shown in **(C′)**. Note that many BDA labeled neurons are observed in L5 of A29, A30 and V2M **(A)** with a few in V1 **(A)** and V2L (arrowheads in **G**). Bars: 500 μm in **(A)** for panels **(A,C,G)**; 300 μm in **(B)** for panels **(B,E,H,I)**.

Brain-wide distribution of anterogradely labeled axon terminals was also examined following BDA injections into A23 ~ of the rats (4 cases). For example, one BDA injection restricted in A23 ~ (Figure 15A) leads to terminal labeling mainly in L1 and L5 of ORBm (Figure 15B), L1 and L3 of ACA (Figures 15C,C’), AM (Figure 15D), Cla (Figure 15E), LD (Figure 15F), L3 of the PrS-PoS (Figure 15G), and L5-6 of the PoR (Figure 15H). Interestingly, like the distribution of the neurons projecting to A23~, full A-P extent of the medial part of the LP-Pul receives strong inputs from A23 ~ (Figures 14B,D,F,H). Weak terminal labeling is also detected in L5-6 of the V2L (Figure 15I), L1 of the PL and in the M2, MEC, pretectal nucleus (PTN), superior colliculus (SC), dorsomedial CPu, zona incerta (ZI), anterolateral portion of the pontine nucleus (PN) and the PtA.

### Efferent projections of A23 ~ in the mice

**Figure 16:**
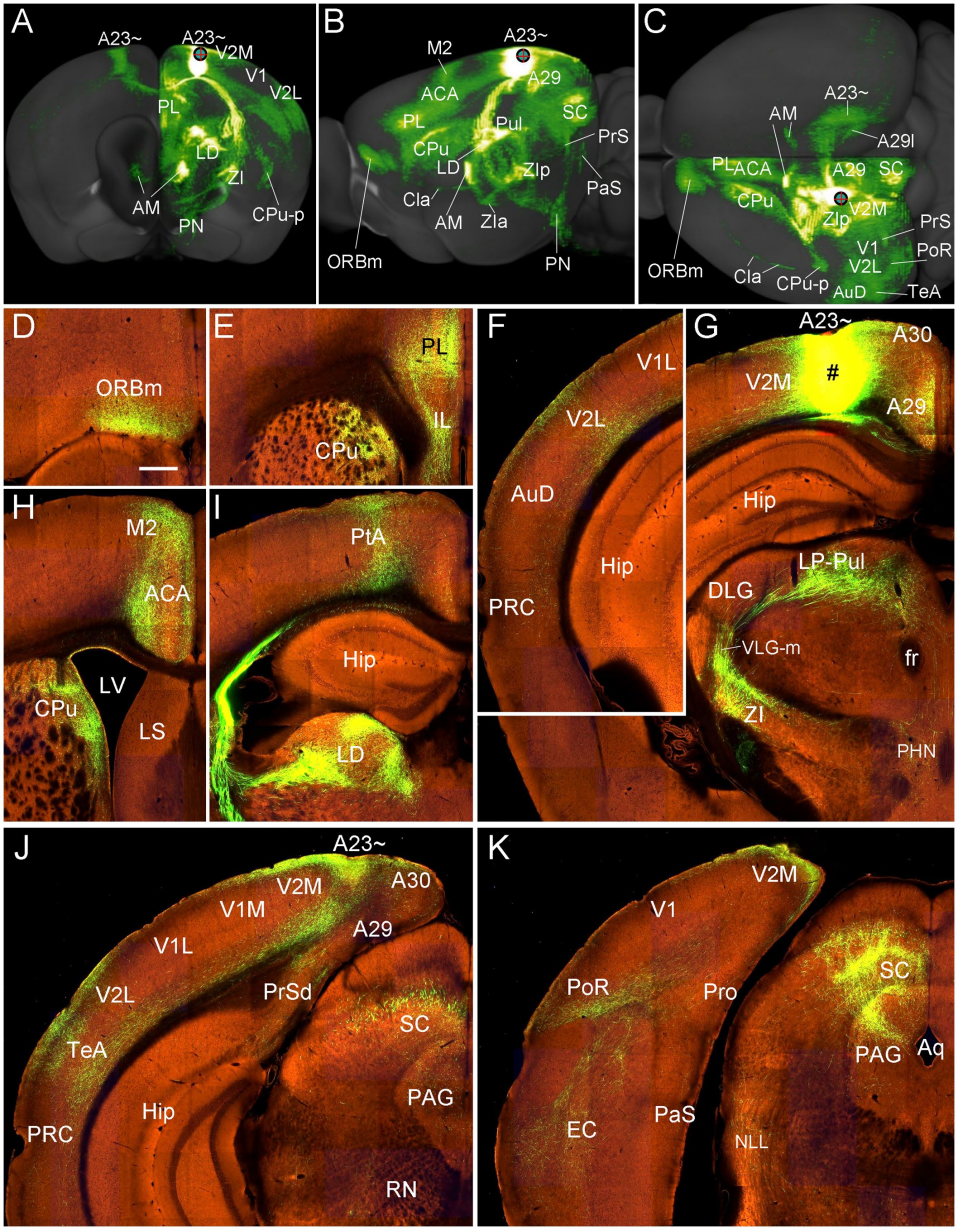
Efferent projections of A23 ~ revealed with the viral tracers in the mouse. **(A–C)** Overall projection pattern of the A23 projections in a *Emx1*-Cre mouse (experiment ID: 562674923) on the anterior **(A)**, lateral **(B)** and dorsal **(C)** aspects of the brain. Some major target regions are marked. Note the obvious terminal labeling in the ipsi- and contralateral AM and A23 ~ **(A,C)**. **(D–I)**. Labeled axon terminals in the ORBm (D), PL and CPu (E, H), V2L (F), LP-Pul and ZI (G), ACA and M2 **(H)**, PtA and LD **(I)**, posterior TeA and A23 ~ **(J)**, and in PoR, EC, SC and PAG **(K)**. The injection site is shown in **(G)**. For orientation of the sections in panels **(D–K)**, medial is at the right and dorsal is at the top. Bar: 400 μm in **(D)** for panels **(D–K)**.

To further confirm the efferent projections of A23 ~ using a more sensitive tracing method we searched the Allen connectivity dataset for the cases with viral tracer injections in A23 ~ (4 cases; experimental IDs: 593018150, 576341623, 575782182 and 562674923). For example, as shown in Figure 16, the injection in the anterior A23 ~ of the mouse (see injection site in Figures 16A–C,G) produces strong axon terminal labeling in both ipsi- and contra-lateral A23~, A29l and AM (Figures 16A,C) and many ipsilateral structures (Figures 16A–K). The latter structures include the ORBm (Figures 16B–D), PL and CPu (Figures 16B,C,E), V2L (Figures 16A,C,F), A29, VLG-m and ZI (Figures 16A–C,G), LP-Pul (Figures 16B,G), ACA and M2 (Figures 16B,C,H), LD and PtA (Figures 16B,I), V2M, posterior TeA and A23 ~ (Figures 16C,J), PoR and MEC (Figures 16C,K), nucleus of lateral lemniscus (NLL), SC and PAG (Figures 16B,C,K). Labeled axon terminals are also seen in the posterior CPu (CPu-p), anterolateral pontine nucleus (PN), Cla, PrS-PoS, and PaS (Figures 16A–C) as well as in the deep layers of the PRC, posterior hypothalamic nucleus (PHN), ventral anterior thalamic nucleus (VA), VM, lateral dorsal tegmental area (LDTg), pontine reticular formation (PnRF), paramedial raphe nucleus (PMnR) and midline thalamic nuclei (MiN) such as the central lateral nucleus of the thalamus (CL) and Re.

### Brain-wide connections of A30 in the rats

**Figure 17:**
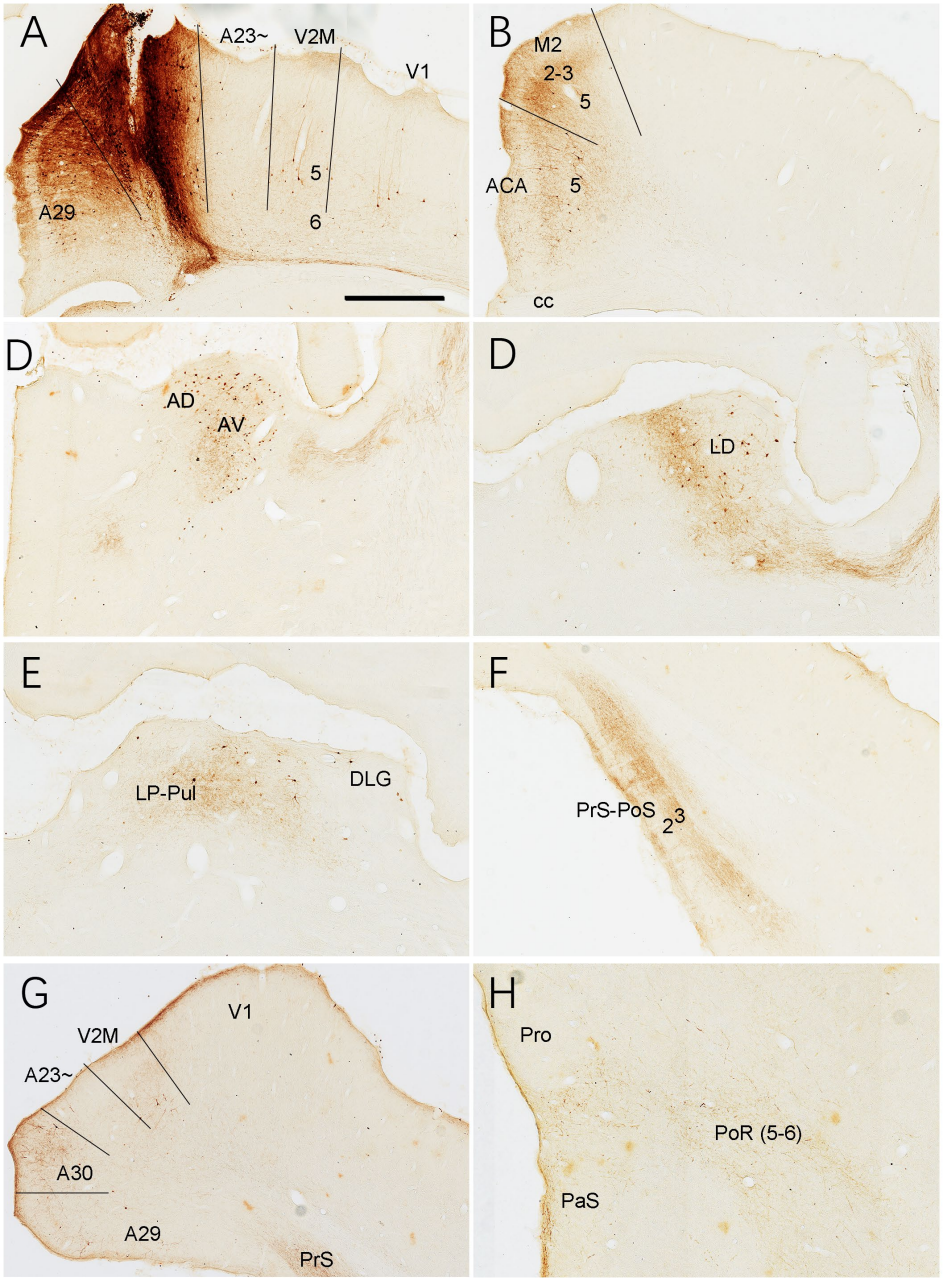
Connections of A30 revealed with BDA in the rat. **(A)** One BDA injection site (#) in A30. Note that BDA labeled neurons are observed in A29, V2M and V1. **(B–H)** BDA labeled neurons and axon terminals in the representative regions. Note the BDA labeled neurons in ACA **(B)**, AD and AV **(C)**, LD **(D)**, LP-Pul **(E)**, V2M and the caudal A30 **(G)**. BDA labeled axon terminals are detected in A29 **(A)**, ACA and M2 **(B)**, AV **(C)**, LD **(D)**, LP-Pul **(E)**, PrS-PoS **(F)**, V2M and posterior A30 **(G)**, and in PaS and PoR **(H)**. Bar: 300 μm in **(A)** for all panels.

As a comparison with A23~, brain-wide connections of A30 were also examined in the rats (3 cases). For instance, following one BDA injection in A30, some retrogradely labeled neurons are found to distribute in L5 of A29, V2M, V1 and ACA (Figures 17A,B), AD and AV (Figure 17C), LD (Figure 17D), LP-Pul (Figure 17E), posterior A30 and posterior V2M (Figure 17G), Cla, V2L and M2. As seen in the same case, BDA labeled axon terminals are detected in A29 (in L1 and L5; Figure 17A), ACA (in L1 and L3; Figure 17B), M2 (in L1 and L2-3; Figure 17B), AV (Figure 17C), LD and RT (Figure 17D), LP-Pul (Figure 17E), PrS-PoS (in L1 and L3; Figure 17F), posterior V2M and A30 (in L2-3; Figure 17G), L1 of the PaS and L5-6 of the PoR (weak labeling in Figure 17H), L1 and L6 of the V1 and V2L (weak labeling), SC, PTN, ZI, PN, and CPu. Compared to BDA injections, FG injections in A30 labeled much more neurons in the regions containing BDA labeled neurons.

## Discussion

Since Brodmann’s comparative cortical mapping, the PCC (mainly A23) has been treated as a unique structure in human and NHP brains. Lower animals such as rabbits and rodents were not reported to have the equivalent of the primate PCC based on cytoarchitectonic criteria (Brodmann, 1909; Swanson, 2004; Vogt et al., 2005; Sugar et al., 2011; Wang et al., 2020). However, functional studies suggest that the PCC is an important component of the default mode network (DMN), which is reported in both primates and rodents (Mantini et al., 2011; Lu et al., 2012; Stafford et al., 2014; Hsu et al., 2016). The DMN (including the PCC) in human brains is heavily involved in autobiographical information, self-reference, thinking about others, spatial episodic memory, and plan for future (Andrews-Hanna, 2010; Leech and Sharp, 2014; Rolls, 2019). These brain functions are basic ones and thus the PCC (mainly A23) should also exist in the rodents. The present study has revealed the possible rodent equivalent of the primate A23 in both rats and mice based on topographical relationship with A30, conservative AM inputs to A23, the differential expression patterns of the genes (*Tshz2, Penk, Cux2,* and *Igfbp5*) and connections between the RSagl (roughly corresponding to A23~) and A30 of the mice (Hu et al., 2020; Lu et al., 2020; the present study) and similar brain-wide afferent and efferent connections of rodent A23 ~ and primate A23. Since we cannot distinguish A23 from A31, and A31 is probably very small in the rodents (if it exists), we have termed the possible rodent equivalent of the PCC as A23 ~ in this study. One of the most important findings of the present study is that A23 ~ in the rodents mainly interconnect with multimodal sensory cortical and subcortical regions. The former regions include ORBm, ACA, medial PtA/A7, PoR, RS, part of V2L, part of M2 and posterior TeA while the latter includes AM, LP-Pul, LD, CPu, PTN, SC and Cla. Finally, our findings also suggest that the feature of strong AM innervation to A23 could be used to consistently distinguish A23 from adjoining areas across species.

### Anteromedial thalamic inputs define possible rodent cingulate cortical area 23

The present study has defined the location and extent of the possible rodent equivalent of the primate A23 (A23~). Like in the primates, A23 ~ in both rats and mice receives its main inputs from the AM rather than from the AD and AV. In contrast, the adjoining A30 receives its main inputs from the AD and AV rather than from the AM. The terminal field in A23 ~ identified with the AM inputs roughly occupies the region previously treated as retrosplenial area 29d (Shibata, 1993), RSagl (Swanson, 2004), a part of the RSag (Sripanidkulchai and Wyss, 1986; Wyss and van Groen, 1992) or as visual area V2MM (Paxinos and Franklin, 2012; Paxinos and Watson, 2013) or area 18b (Miller and Vogt, 1984; van Groen et al., 1999). Specifically, in coronal sections, A23 ~ in the rats and mice forms an A-P band lateral to A30. This finding of AM projections to A23 (but not to A30) is consistent with that reported in primates (Baleydier and Mauguiere, 1980; Vogt et al., 1987; Shibata and Yukie, 2003). For example, when the retrograde tracers were restricted to A23 of the monkeys, the labeled neurons were mostly observed in the AM with no or few in the AD and AV (Baleydier and Mauguiere, 1980; Vogt et al., 1987; see also cases M-2-WGA-HRP and M-5-CTb in Shibata and Yukie, 2003). However, when the tracers were involved in adjoining A30, labeled neurons could also be found in the AD and AV in addition to the AM (see case M1-WGA-HRP in Shibata and Yukie, 2003; Buckwalter et al., 2008). In contrast, when the tracers were restricted in A30 and A29 of the monkeys, many labeled neurons were seen in the AD and AV with no or few in A23 (see cases M3-CTb and M5-DY in Shibata and Yukie, 2003).

### Comparative connections of area 23 in primates and rodents

The relative size of A23 in NHP and human brains is much larger compared to A29, and this A23 can be further divided into three subdivisions (A23a, b, and c). In sharp contrast, the relative size of A23 ~ in the rodents is much smaller in comparison with A29. Despite this difference, the major afferent and efferent connections of A23 in the rodents and primates appear similar and comparable. For example, the primate A23 is reported to receive afferents from the AM, Pulm, Cla, dorsolateral frontal cortex (DFC; A9, A10, and A46), ORBm (A11, A13, and A14), ACC (A24 and A32), RS (A29 and A30), parahippocampal cortex (PHC; areas TH, TL, and TF), posteromedial inferior parietal cortex (areas Opt and PGm), posterior superior temporal cortex (areas TAc and Tpt) and dorsal bank of the superior temporal sulcus (area TPO) (Baleydier and Mauguiere, 1980, 1985; Vogt et al., 1987; Van Hoesen et al., 1993; Yukie, 1995; Kobayashi and Amaral, 2003; Shibata and Yukie, 2003; Buckwalter et al., 2008; Seltzer and Pandya, 2009). Similarly, the rat A23 ~ receives its main inputs from the AM, LP-Pul (like monkey LP and Pul), Cla, a part of M2 (likely similar to A24c), ORBm, ACA (similar to A24ab), A29-30, PoR (similar to PHC), medial PtA (similar to area Opt/PGm), a part of V2L (similar to area TPO) and the posterior TeA (similar to area TAc/Tpt).

The efferent targets of the monkey A23 include the AM, SC, PTN, PN, Cla, LD, LP, Pulm, CPu, PrS, A29-30, ACC, PHC, ORBm, DFC, areas TPO and TAc/Tpt (Pandya et al., 1981; Van Hoesen et al., 1993; Shibata and Yukie, 2003; Parvizi et al., 2006; Kobayashi and Amaral, 2007; Seltzer and Pandya, 2009). These are also similar to the target regions in rodents such as the AM, SC, PTN, PN, Cla, LD, LP-Pul, CPu, PrS, A29-30, ACA, PoR, ORBm, a part of M2, a part of V2L and the posterior TeA (e.g., Figure 15). However, the rodent equivalent of the monkey DFC remains to be identified.

### Comparison of area 23 ~ with areas 30 and 29 in rodents

A29 in the rodents is easily distinguishable from A30 due to its unique outer granular layer (L2-3). However, the border between rodent A30 and A23 ~ is difficult to be identified, particularly in Nissl-stained sections since rodent A23 ~ does not display a thin inner granular L4 while the primate A23 does. This is the major reason why A23 ~ was not identified in previous studies. However, given the primate cortex having overall much more granular cells in L4 compared to the rodent cortex, it is not very surprising to see few granular cells in L4 of the rodent A23~. In literature, significant differences in cytoarchitecture are frequently observed between the primate and rodent brains. For example, hippocampal CA1 cells in NHP and human brains are lightly stained and loosely packed while those in the rats and mice are darkly stained and very densely packed (Ding, 2013). However, the topographic relationship of CA1 with CA2/CA3 and ProS/Sub as well as the connectional patterns of these hippocampal subfields remain similar across species (Ding, 2013; Ding et al., 2020). The present study has provided a connectional approach that enables the differentiation between A30 and A23. The AM inputs predominantly terminate in A23 ~ with few in adjoining A30 and V2M while the inputs from the AD and AV mostly innervate A29 with fewer in A30 and almost no in A23~. The extent and boundaries of A30 in the mice identified in this study are consistent with those identified with molecular markers such as *Penk* (Hu et al., 2020), *Ddit4l, Npnt, Fam3c* and *Vamp1*, and are distinct from A23 ~ (Figure 2). It is obvious that the rats and mice have a relatively huge A29 and much smaller A30 and A23~. The connectivity of A29, A30 and A23 ~ in the rodents is different from one another. For example, A29 receives its inputs mainly from the AD, AV, LD, Cla, ACA (A24), A30, PrS and Sub (Wyss and van Groen, 1992; Ding et al., 2020) and projects mainly to the AD, AV, LD, A24, A23~, PrS-PoS and PaS (Wyss and van Groen, 1992; Ding, 2013). A30 gets its inputs mainly from the LD, AD, AV, M2, V2M, V1, V2L and PoR, (van Groen and Wyss, 1992; also see https://connectivity.brain-map.org) and innervates the LD, A29, visual (V2M, V2L and V1) and auditory (A2 and the posterior TeA) cortices as well as the PrS-PoS, PaS and PoR (Wyss and van Groen, 1992; Ding, 2013; the present study). A23 ~ in the rodents obtains its inputs mainly from the AM, LP-Pul, A29, ACA and LD, and mainly targets the AM, LP-Pul, ACA, Orbm, PrS-PoS, SC (this study). Taken together, A29, A30 and A23 ~ have differential molecular signature and connectivity and thus likely play different roles in spatial processing, navigation, and adaptive behaviors.

### Comparison of area 23 with parietal association cortex

Since A23 also adjoins medial parietal association cortex in both monkeys and rodents, it could be important to explore whether some similarity and difference in the connectivity exits between the two areas. In the primates, the PtA or area 7 (A7) mainly contains areas 7a (PG), 7b (PF), 7ip, 7 m (PGm) and Opt (Leichnetz, 2001; Seltzer and Pandya, 2009). First, A23 is strongly innervated by AM inputs while, PtA did not receive significant AM inputs (Schmahmann and Pandya, 1990; Leichnetz, 2001; Buckwalter et al., 2008). Second, PtA appears to send strong projections to A30 while A23 has strong connections with A29 (Mesulam et al., 1977; Cavada and Goldman-Rakic, 1989; Kobayashi and Amaral, 2003). Third, PtA in general has stronger outputs to the PrS than A23 does (Seltzer and Van Hoesen, 1979; Pandya et al., 1981; Ding et al., 2000). Fourth, A23 and PtA display stronger (Van Hoesen et al., 1993; Parvizi et al., 2006) and weaker (Weber and Yin, 1984; Baizer et al., 1993) projections to the Cla, respectively. All these differences also appear true for rodents. First, the PtA does not appear to receive the AM inputs in rats (Reep et al., 1994; Torrealba and Valdés, 2008; Olsen and Witter, 2016) while A23 ~ does (the present study). Second, the PtA and A23 ~ have stronger connections with A30 and A29, respectively (Wilber et al., 2014; the present study). Third, the PtA and A23 ~ send stronger and weaker projections to the PrS, respectively (Ding, 2013; Olsen et al., 2017; the present study). Fourth, the PtA and A23 ~ have weaker and stronger connections with Cla, respectively (Lyamzin and Benucci, 2019; the present study).

### Functional consideration of area 23

Since A23 was not reported in the rodents, previous functional studies of A23 or PCC were mainly conducted in human and NHP brains. Briefly, A23 is a key component of the DMN and displays increased activity when retrieving episodic and autobiographical memories, planning for future, understanding the behavior and thoughts of others, appraising emotional information and “freewheeling” during unconstrained “rest” (Leech and Sharp, 2014; Rolls, 2019; Zhang and Volkow, 2019). In addition, A23 is sensitive to the changes in arousal state, awareness and the environment and participates in controlling the balance between internal and external attention (Leech and Sharp, 2014; Barack and Platt, 2021; Chang et al., 2021). Connectional studies in the NHP appear to support these functions since A23 converges many aspects of the inputs from hetero-modal cortical and subcortical regions.

The monkey A23 receives direct inputs from multimodal association cortices such as visual (areas 18, 19 and TPO), parietal (areas 7a and Opt), auditory (areas TB, TA and Tpt) and parahippocampal (areas TH and TF) association cortices. It is also innervated by different limbic cortices such as the ORBm (areas 11, 13 and 14), ACC (A24, A32 and A25), RS (A29 and A30), PRC (areas 35 and 36) and entorhinal cortex (MEC and LEC) (Baleydier and Mauguiere, 1980; Vogt and Pandya, 1987; Van Hoesen et al., 1993; Yukie, 1995; Parvizi et al., 2006; Seltzer and Pandya, 2009; Balcerek et al., 2021). In addition, the DFC also sends projections to A23 (Vogt and Pandya, 1987; Kobayashi and Amaral, 2003; Parvizi et al., 2006). Most of these connections have been confirmed in the rodent A23 ~ except for the DFC, which remains to be explored. Therefore, A23 has access to a variety of information from different modalities and thus is a critical hub for integration and regulation of different information.

Other important information also reaches A23. For example, the ORBm in both monkeys and rodents displays strong reciprocal connections with A23 (Vogt and Pandya, 1987; Van Hoesen et al., 1993; Parvizi et al., 2006; the present study). Specialized areas for value updating (area 13) and goal selections (area 11) were reported in monkey ORBm (Murray et al., 2015) and this may be also true for human ORBm (Saez et al., 2018). The ORBm may be a critical site for temporal cognition, integrating reward magnitude and delays, which are needed for value processing (Sosa et al., 2021). The value-related information in the ORBm would project to and interact with A23 and thus could contribute to the functions of A23 in planning for future and understanding the behavior and thoughts of others.

The RS is another region with heavy connection with A23 in both monkeys (Morris et al., 1999; Kobayashi and Amaral, 2003, 2007) and rodents (the present study). For instance, A29 in the rodents receive its major inputs from the Sub, PrS-PoS and A30 as well as the AD, AV and LD, all of which are heavily involved in spatial processing and navigation (Wyss and van Groen, 1992; sugar et al., 2011; Ding et al., 2020; the present study). A29 mainly projects to the PrS-PoS, A30, PaS, ACA, AD, AV and LD (Wyss and van Groen, 1992) and A23 (the present study). In contrast, A30 appears to converge sensory, motor and spatial information since A30 receives dense inputs from the primary (V1) and secondary (V2M and V2L) visual inputs (Wyss and van Groen, 1992; the present study) as well as from the secondary motor area M2 (the present study), LD, LP-Pul and PtA (Torrealba and Valdés, 2008). A30 projects back to these afferent regions and to the PrS-PoS and A29 (the present study). Although A30 in the rodents does not appear to have strong connections with A23, it heavily projects to A29, which strongly innervate A23. Therefore, A23 could access different spatial information about the environment via the RS and enable its roles in spatial learning and memory such as retrieving episodic and autobiographical memories and adapting changes in the environment. Other aspects of memory-related information could reach A23 via the AM, PoR, LEC and MEC, all of which receive dense projections from the Sub and/or ProS. The latter two are the output structures of the hippocampal memory system (Ding et al., 2020).

The ACC is heavily and reciprocally connected with A23, RS, ORBm, PoR, AM, BL and Cla (Baleydier and Mauguiere, 1980; Vogt et al., 1987; Vogt and Pandya, 1987; Van Hoesen et al., 1993; Kobayashi and Amaral, 2003). The ACC probably carries a myriad of signals such as error detection, reinforcement/feedback, value, response conflict, autonomic and emotional states, which are necessary for the modulation of attention and task-relevant/irrelevant signals so that difficult decisions can be made and action plans adapted when necessary (Rolls, 2019; Brockett and Roesch, 2021; Seamans and Floresco, 2022). All these information can reach to and interact with A23 via the strong reciprocal connections between A23 and ACC (Baleydier and Mauguiere, 1980; Van Hoesen et al., 1993; Parvizi et al., 2006; Rolls, 2019; the present study).

Finally, A23 in both primates and rodents is also reciprocally and heavily connected with the Cla (Baleydier and Mauguiere, 1980; Van Hoesen et al., 1993; Parvizi et al., 2006; the present study). Many previous studies reported that the Cla is heavily involved in attention, awake-sleep states, sensory and salience processing likely via regulation of cortical excitability (see review in Smith et al., 2020). Recent works indicate that Cla mainly exerts inhibitory roles on widespread cortical regions including cingulate cortex (Smith et al., 2020). Therefore, as an example, it is likely that the Cla could inhibit or interacts with the DMN (including A24, A23 and others) when salient stimuli appear in the environment so that the focus of attention can be switched to more salient tasks.

In summary, the PCC (A23) is a critical hub for the integration and modulation of multimodal sensory information underlying spatial processing, self-reflection, attention, plan for future and many adaptive behaviors.

## Data availability statement

The original contributions presented in the study are included in the article/supplementary material, further inquiries can be directed to the corresponding author.

## Author contributions

S-LD: conceptualization. X-JX, S-LD, S-QC, C-HC, S-YZ, and H-RC: investigation. X-JX and S-LD: manuscript writing. S-LD, S-QC, and X-QZ: supervision. All authors read and approved the final manuscript.

## Funding

This work was partially supported by grants from the National Natural Science Foundation of China (No. 31771327) and the Guangzhou Science Technology Plan Project (No. 202206060004).

## Conflict of interest

The authors declare that the research was conducted in the absence of any commercial or financial relationships that could be construed as a potential conflict of interest.

## Publisher’s note

All claims expressed in this article are solely those of the authors and do not necessarily represent those of their affiliated organizations, or those of the publisher, the editors and the reviewers. Any product that may be evaluated in this article, or claim that may be made by its manufacturer, is not guaranteed or endorsed by the publisher.

## Figures and Tables

**Table 1:** 

3V	Third ventricle
4V	Fourth ventricle
A29	Area 29
A29l	Lateral division of area 29
A29m	Medial division of area 29
A30	Area 30
A23 ~	Rodent equivalent of area 23
A23	Area 23
ac	Anterior commissure
ACA	Anterior cingulate area
AD	Anterodorsal nucleus of thalamus
AM	Anteromedial nucleus of thalamus
Aq	Aqueduct
AuD	Dorsal auditory association cortex
AV	Anteroventral nucleus of thalamus
BL	Basolateral amygdaloid nucleus
cc	Corpus callosum
ccs	Splenium of corpus callosum
cla	Claustrum
CPu	Caudate putamen
CPu-p	Posterior part of caudate putamen
DFC	Dorsolateral prefrontal cortex
DLG	Dorsal lateral geniculate nucleus
EC	Entorhinal cortex
fr	Fasciculus retroflexus
Hip	Hippocampal formation
IG	Indusium griseum
IL	Intralimbic cortex
LD	Lateral dorsal nucleus of thalamus
LEC	Lateral entorhinal cortex
LP-Pul	Lateroposterior nucleus-pulvinar complex
LS	Lateral septal nucleus
LV	Lateral ventricle
M2	Secondary motor cortex
MEC	Medial entorhinal cortex
NAC	Nucleus accumbens
NC	Nucleus coeruleus
NHP	Non-human primate
NLL	Nucleus of lateral lemniscus
ORBm	Medial orbitofrontal cortex
PAG	Periaqueductal gray
PaS	Parasubiculum
PHC	Posterior parahippocampal cortex
PHN	Posterior hypothalamic nucleus
PL	Prelimbic cortex
PN	Pontine nucleus
PoR	Postrhinal cortex
PRC	Perirhinal cortex
Pro	Area prostriata
PrS	Presubiculum
PrSd	Dorsal presubiculum (postsubiculum)
PrSv	Ventral presubiculum
PT	Paratenial nucleus
PtA	Parietal association cortex
PTN	Pretectal nucleus
PV	Paraventricular hypothalamic nucleus
PVT	Paraventricular nucleus of thalamus
Re	Reuniens nucleus
RN	Red nucleus
RS	Retrosplenial cortex
RSag	Agranular retrosplenial cortex
RSagl	Lateral agranular retrosplenial cortex
RSg	Granular retrosplenial cortex
RT	Reticular thalamic nucleus
SC	Superior colliculus
Sd	Dorsal subiculum
smt	Stria medullaris of thalamus
Spo	Polymorphic layer of subiculum
Sub	Subiculum
SuS	Supracallosal subiculum
TeA	Temporal association cortex
V1	Primary visual cortex
V2L	Lateral secondary visual cortex
V2M	Medial secondary visual cortex
VA	Ventroanterior nucleus
VLG	Ventral lateral geniculate nucleus
VLG-m	Medial part of VLG
wm	White matter
ZI	Zona incerta
ZIa	Posterior part of zona incerta
ZIa	Anterior part of zona incerta

## Footnotes

^1^ https://www.blueprintnhpatlas.org/

^2^ https://help.brain-map.org/display/nhp/Documentation

^3^ https://mouse.brain-map.org/

^4^ https://connectivity.brain-map.org/
